# Convergence of neuroinflammation across major neurotropic viral exposomes in AD and ADRD

**DOI:** 10.1186/s12974-026-03876-2

**Published:** 2026-05-29

**Authors:** Jamile Harmouch, Ryan Green, Karthick Mayilsamy, Kristina Tosi, Subhra Mohapatra, Shyam S Mohapatra

**Affiliations:** 1 Department of Internal Medicine and Institute of Translational Virology and Innovation, University of South Florida Morsani College of Medicine, Tampa, FL 33612 USA; 2 Department of Molecular Medicine, University of South Florida Morsani College of Medicine, Tampa, FL 33612 USA; 3 James A Haley VA Hospital, Research Service, Tampa, FL 33612 USA

**Keywords:** Neurotropic viruses, Neuroinflammation, Infectious brain hypothesis, Alzheimer’s disease, Amyloid-β, Tau hyperphosphorylation, Blood-brain barrier, APOE4

## Abstract

**Background:**

Alzheimer’s disease (AD) and Alzheimer’s disease–related dementias (ADRD) are multifactorial neurodegenerative disorders driven by complex interactions among genetic susceptibility, aging, and environmental exposures. Growing epidemiological and mechanistic evidence implicates neurotropic viral exposomes, defined as cumulative lifetime viral infections, as significant contributors to AD risk. Viral encephalitis and common viral infections, including herpes simplex virus type 1 (HSV-1), human immunodeficiency virus (HIV), cytomegalovirus (CMV), SARS-CoV-2, and influenza, have been associated with an increased incidence of AD/ADRD; however, the molecular mechanisms underlying these associations remain incompletely understood.

**Methods:**

A systematic literature review was conducted using PubMed, Web of Science, Scopus, and Google Scholar (1990–2025) to identify epidemiological, experimental, and mechanistic studies linking viral infections to AD-related pathology. Systems biology approaches were applied using Cytoscape, STRING, KEGG, WikiPathways, and Ingenuity Pathway Analysis to construct protein–protein interaction networks and identify convergent biological processes shared between AD and viral host-response pathways. Functional enrichment analyses focused on neuroinflammation, amyloid-β (Aβ) metabolism, tau pathology, autophagy, and blood–brain barrier (BBB) integrity.

**Results:**

Across diverse viral infections, strong convergence was observed in innate immune activation pathways, including microglial priming and NLRP3 inflammasome signaling, accompanied by chronic production of proinflammatory cytokines (IL-1β, TNF-α, IFN-γ). Multiple viruses modulated amyloidogenic APP processing, impaired Aβ clearance, promoted tau hyperphosphorylation, disrupted autophagy-lysosomal systems, and compromised BBB integrity. Systems-level analyses revealed overlapping signaling hubs, including NF-κB, MAPK, PI3K-Akt, and cGAS-STING that amplify neurodegenerative cascades, with effects most pronounced in genetically susceptible populations such as APOE4 carriers.

**Conclusions:**

Collectively, current evidence supports a mechanistic link between viral exposomes and AD/ADRD mediated through convergent neuroinflammatory, and proteostatic pathways. Although viral infections alone are unlikely to be sufficient to cause AD, recurrent or persistent viral exposures may act as potent disease modifiers that accelerate neurodegenerative processes. Integrating viral biomarkers, genetic risk stratification, and systems biology approaches offers promising opportunities for early diagnosis, prevention, and development of mechanism-guided therapeutic strategies.

**Supplementary Information:**

The online version contains supplementary material available at 10.1186/s12974-026-03876-2.

## Introduction

Neurodegenerative diseases, characterized by neuronal loss and cognitive impairment, represent a major global health challenge with steadily increasing incidence [1]. Among them, Alzheimer’s disease (AD) is the most prevalent, followed by Parkinson’s disease (PD) [1]. The global prevalence of PD exceeds 6 million and is projected to reach 25.2 million cases by 2050 [2, 3] In contrast, AD affects an estimated 6.9 million individuals in the U.S alone, with cases expected to rise to 13.8 million by 2060 [4], underscoring its comparatively greater public health burden. AD accounts for approximately 60–80% of all dementia cases [5].

**Fig. 1:**
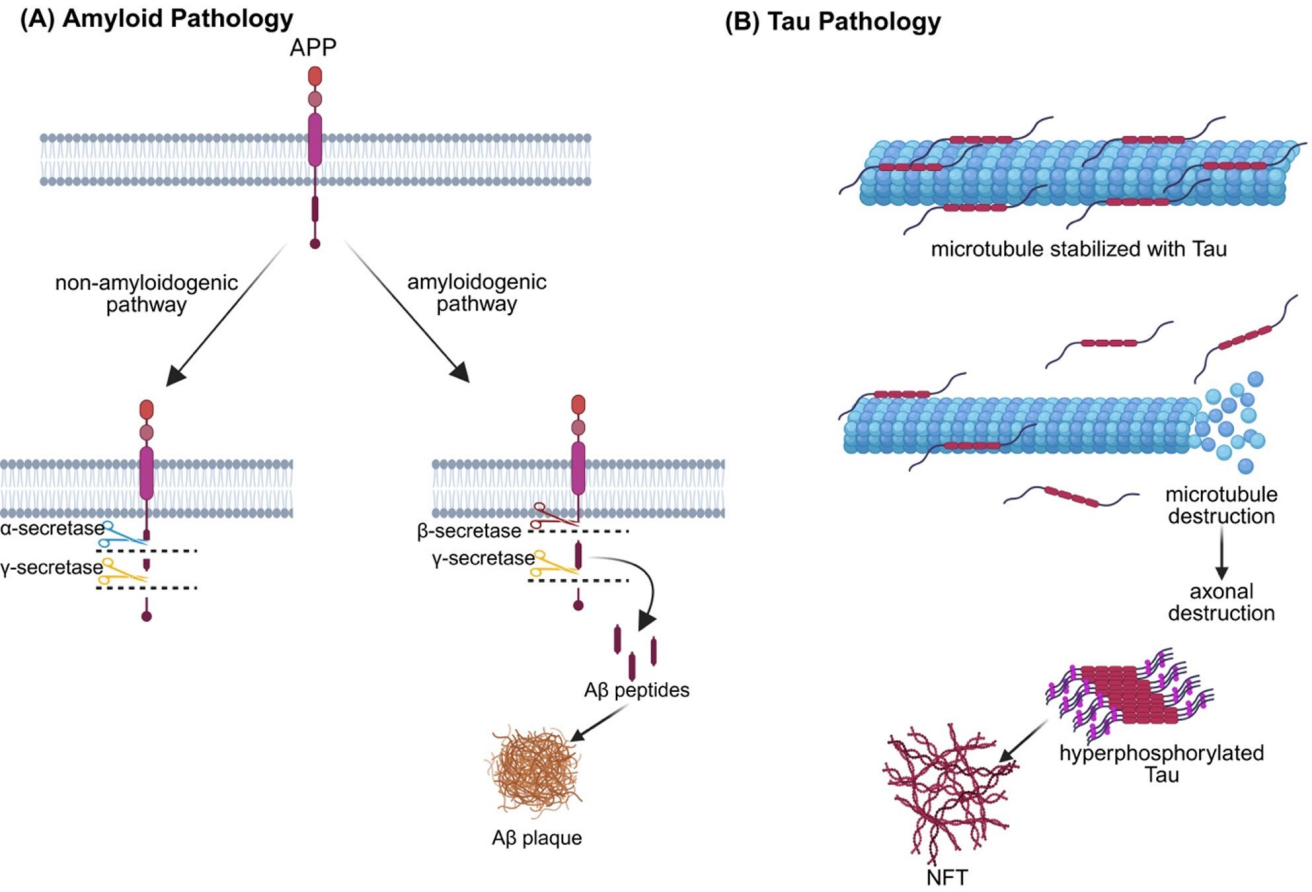
APP Processing and Tau Pathology in Alzheimer’s Disease. **A **Amyloid Precursor Protein (APP) can be processed by two metabolic pathways. The protective non-amyloidogenic pathway involves cleavage by α-secretase and γ-secretase of APP within the Aβ domain preventing Aβ formation. The pathogenic amyloidogenic pathway involves the cleavage by β-secretase and γ-secretase, generating Aβ monomers that aggregate into plaques. **B** Tau proteins normally stabilize microtubules. Tau pathology is characterized by their hyperphosphorylation causing their detachment, leading to microtubule destabilization and neurofibrillary tangles (NFT) formation, both of which lead to neuronal death

Pathologically, AD is defined by extracellular amyloid-β (Aβ) plaques and intracellular neurofibrillary tangles (NFTs) composed of hyperphosphorylated Tau protein [6]. These lesions contribute to synaptic loss, neuroinflammation, and neuronal degeneration. Aβ overproduction and accumulation originate from dysregulated amyloid precursor protein (APP) processing, with increased APP levels and a pathogenic shift towards the β-secretase amyloidogenic pathway mediated by the protease BACE1 (β-site APP cleaving enzyme 1) [7]. Aggregated Aβ forms extracellular amyloid fibrils and plaques, which can appear as dense-core or diffuse lesions, with dense plaques correlating with more advanced AD pathology [8, 9] Tau pathology also plays a central role. Hyperphosphorylated Tau disrupts microtubule stability, leading to cytoskeletal collapse and neuronal death (Fig. 1). Aβ interacts synergistically with Tau by activating PAX6 and GSK-3β signaling, promoting Tau hyperphosphorylation, synaptic mis-localization and neurotoxicity [10, 11]. Aβ plaques further increase soluble phosphorylated Tau (p-Tau), accelerating formation of insoluble Tau aggregates that spread trans-synaptically and directly contribute to neuronal loss and cognitive decline [12]. Together, these processes support a model in which Aβ acts as an initiating factor, with Tau pathology driving downstream disease progression [13].

Neuroinflammation is not merely a secondary response but a major driver of AD pathogenesis. Activated microglia and astrocytes respond to Aβ aggregates, Tau pathology, and infectious triggers by releasing pro-inflammatory cytokines including IL-1β, IFN-γ, TNF-α, that impair neuronal function. The cytokines also elevate oxidative stress through increased inducible nitric oxide synthase (iNOS) activity, and nitric oxide (NO) production, amplifying neurotoxicity and Aβ aggregation. Activated microglia and Aβ also trigger caspases pathways (caspase-1, caspase-3) and the NLRP3 inflammasome, further promoting neurodegeneration [14]. Blood-brain barrier (BBB) dysfunction is another key feature of AD and correlates with disease severity. BBB breakdown promotes neuroinflammation and oxidative stress, enhancing Aβ accumulation. Conversely, Aβ and pro-inflammatory cytokines exacerbate BBB disruption, creating a self-reinforcing cycle that accelerates disease progression [15]. Despite identification of these hallmark processes, the initial triggers of AD remain incompletely understood [16].

While the amyloid cascade hypothesis, proposing Aβ as the central pathogenic driver, remains influential, it faces increasing scrutiny [17] Anti-Aβ antibodies such as Solanezumab have lowered Aβ levels without producing meaningful cognitive improvement [18], thus challenging this hypothesis as a complete explanation for AD. This has shifted attention toward alternative or complementary models. One such model, the Infectious Brain hypothesis, proposes that microbial infections and subsequent chronic neuroinflammation act as triggers for neurodegenerative diseases, including AD [19, 20]. Multiple infectious agents including viruses, bacteria such as *Chlamydia pneumoniae* (CP), *Helicobacterpylori* (HP), *Porphyromonas gingivalis* (*P. gingivalis*), Spirochetes and eukaryotic unicellular parasites (e.g., *Toxoplasma gondii*), have been linked to AD due to their ability to activate innate immunity, induce inflammation, and increase oxidative stress, thereby contributing to cognitive impairment and AD pathology.

Recent large-scale association studies have revealed strong links between viral infections and increased risk for multiple neurodegenerative diseases (NDDs), including AD, amyotrophic lateral sclerosis (ALS), generalized dementia (DEM), vascular dementia (VAS), Parkinson’s disease (PD), and multiple sclerosis (MS). Leveraging national biobank data from FinnGen and UK Biobank, investigators identified and replicated numerous significant associations between prior viral exposures and subsequent NDD onset. In FinnGen, 45 significant virus-NDD associations were detected, 22 of which were replicated in the UK Biobank. The strongest association was observed between viral encephalitis and AD, with hazard ratios exceeding 30 in some cohorts. Influenza with pneumonia, herpes simplex virus (HSV), varicella-zoster virus (VZV), and several enteric viral infections were also consistently associated with elevated risk for AD and ADRD [21]. Notably, many of these associations persisted for up to 15 years prior to diagnosis, suggesting that viral infections may contribute to chronic neuroinflammatory states and reduced cognitive resilience rather than representing short-term triggers. Importantly, no viral exposure demonstrated a protective effect.

Despite the robustness of these associations, several methodological and practical limitations must be considered. Most studies rely on hospital billing codes and electronic health records rather than laboratory-confirmed infections, increasing the potential for exposure misclassification and limiting mechanistic specificity. The predominance of European ancestry in major biobanks restricts generalizability to other populations. Temporal analyses are also constrained by the relatively recent digitization of electronic medical records, limiting evaluation of early-life infections and lifetime risk trajectories. Reverse causality remains a concern because preclinical neurodegeneration may increase susceptibility to viral infections. Confounding variables including lifestyle, socioeconomic status, comorbidities, and healthcare utilization can influence observed associations. Hospitalization following viral infection may also increase the likelihood of early detection of neurodegenerative condition. Furthermore, severity, recurrence, and subtype of viral exposures are often underreported, and most association studies do not explore the molecular pathways linking viral infection to NDD risk. These limitations underscore the need for more granular, diverse, and mechanistically focused research using laboratory-confirmed viral-exposure data alongside integrated molecular diagnostics to clarify the causal role of neurotropic viruses in AD and ADRD.

Herein, we evaluate the molecular mechanisms by which major viral exposomes activate immune pathways and contribute to the development of neurodegenerative diseases, demonstrating that neuroinflammation represents the central mechanism underlying the ‘infectious brain hypothesis. We focus on selected neurotropic viruses, including HSV-1, HHV6, HHV7, EBV, HIV, CMV, SARS-CoV-2, influenza, RSV, ZIKV and VZV. This review highlights shared mechanistic features between viral host responses and AD pathways using bioinformatics approaches.

## Methods

### Literature search

The literature search was conducted between June and October 2025 using PubMed, Google Scholar, Web of Science, and Scopus. Search terms included: “Alzheimer’s disease”, “Microbial hypothesis”. “Infectious hypothesis”, “neurotropic viruses”, “amyloid- β”, “tau”, “neurodegeneration”, “neuroinflammation”, “blood-brain barrier disruption”, “dementia”, “HSV”, “CMV”, “HIV”, “SARS-CoV-2”, and “influenza”. Boolean strings (AND/OR) were applied to expand or narrow search results. Searches covered peer-reviewed original studies, meta-analyses, and review articles published in English between 1990 and 2025. Reference lists of relevant articles were screened for additional citations (See Supplementary Table 1). Studies focusing on molecular mechanism, host-virus interactions, epidemiological associations, neuropathological outcomes, and methodological limitations were prioritized.

### Bioinformatic analyses

To investigate the molecular/functional commonalities between Alzheimer’s disease and viral infections we utilized the STRING app’s “Disease” search function within Cytoscape to retrieve the top 2,000 Alzheimer’s associated proteins (DOID:10652, confidence score cutoff 0.7) and imported them into a protein-protein interaction network (PPI). This network was merged with AD associated protein lists obtained from the KEGG database (map05010) and the wiki pathways database (WP5124) to generate a more comprehensive network. Next, we used the STRING: Enrichment app in Cytoscape to retrieve functionally enriched gene ontology terms for the AD associated protein network. The enrichment table was filtered to retain only the “Biological Process”, and “Molecular Function” GO categories (Overlap cutoff 0.5, FDR ≤ 0.05). This yielded 4,684 GO terms significantly enriched in the AD PPI network vs. background (whole genome). This procedure was repeated to generate PPI networks and associated enriched GO term lists for eleven viral infections and for the disease process of neuroinflammation. Searches within the STRING: disease app in Cytoscape contained results compiled from multiple databases (STRING, Reactome, WikiPathways, KEGG, Monarch) however, some searches for less well studied viruses did not yield results for all databases (Supplementary Table 1).

Because several viral PPI networks generated using this method contained less than 200 proteins, the networks were supplemented by addition of proteins with evidence of involvement in the viral host response presented in relevant publications by using the “Geneset by Pathway / Process / Disease / Publication” search feature of STRING. Proteins from the following publications describing viral host response were imported (Supplementary Table 1). These publications were selected based on their association with relatively large gene datasets in the STRING search results and their relevance to neurological and/or immune response to the infection by their respective virus. GO term lists enriched in each virus PPI network and for neuroinflammation were computed using the same method previously used to create the AD GO term enrichment. These lists were compared with the AD GO term list in a pairwise fashion using Microsoft Excel and the VLOOKUP function. A table of intersections between enriched GO term lists was created and a chord diagram was used to visualize these intersections (Flourish software, https://flourish.studio). A comprehensive list of AD matching terms for all viruses was compiled and imported into Cytoscape for visualization and network analysis.

Pathway analysis was performed using Ingenuity Pathway Analysis (Qiagen) to explore common molecular signaling pathways modulated by diverse viral infections with evidence of involvement in AD pathological processes. The 83 viral response genes summarized in Supplementary Table 1 were manually added to a new “My Pathway” workspace in IPA to create a custom gene network. These genes were selected based on direct evidence linking them to AD pathology published in peer-reviewed academic journals as discussed throughout this review. Next, the overlay feature was used to query the IPA database for diseases, biological functions, and canonical signaling pathways associated with this network. This feature uses the right-tailed Fisher’s Exact Test to calculate a statistical significance of overlap of the given dataset molecules with various reference sets of molecules that represent annotations such as Canonical Pathways, upstream regulators, diseases, etc. The top disease/function hits along with genes contributing to inflammation of the central nervous system broken down by evidence of their involvement in each individual viral infection.

## Results and discussion

### The ‘infectious brain hypothesis’ revisited

#### Historical emergence

**Fig. 2:**
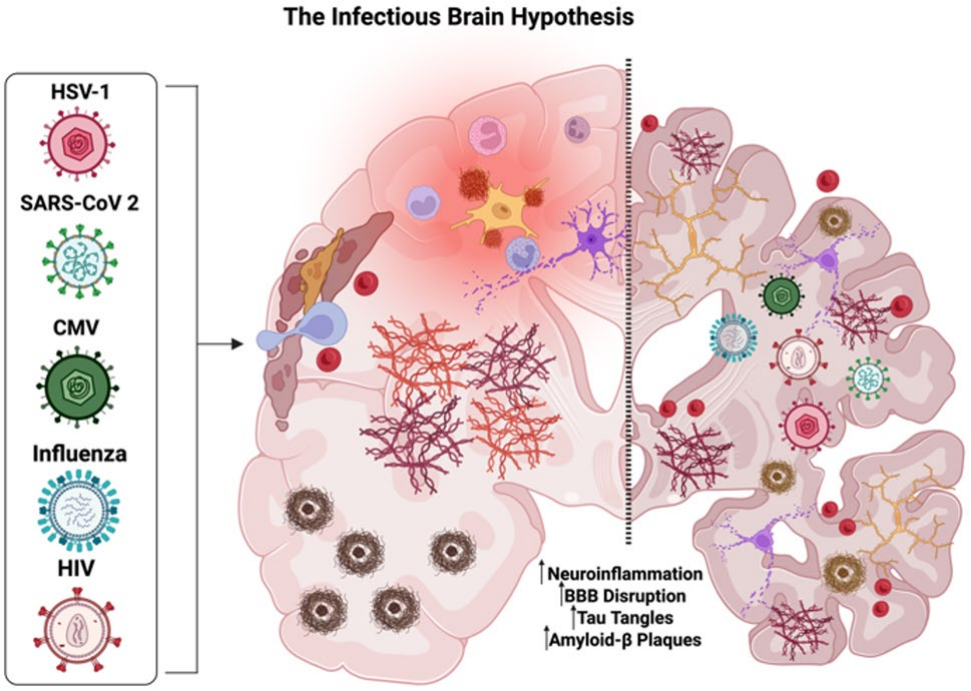
A graphical representation of the ‘Infectious-Inflammatory Brain Hypothesis’

The infectious brain hypothesis has evolved substantially since its early 20th-century origins, when microbial agents were first proposed as potential triggers of neurodegeneration [22]. This hypothesis proposes that microbial infections may play a causative or accelerating role in the development of neurodegenerative diseases (Fig. 2), including AD [23]. Initial support remained limited until a seminal 1991 study identified latent HSV-1 DNA in post-mortem AD brains [24], reinvigorating interest in the contribution of neurotropic pathogens to disease onset and progression. Subsequent investigations confirmed the presence of HSV-1 DNA in both AD and non-AD brains using more sensitive PCR-based methods [24]. Over the following decades, advances in molecular virology and neuropathology broadened the list of implicated pathogens and revealed mechanistic overlap between viral infections and hallmark AD processes, such as Aβ aggregation, Tau hyperphosphorylation, and chronic neuroinflammation [25, 26]. However, early observations were constrained by technical limitations, including inconsistent detection methods, small sample sizes, and difficulty establishing causality, underscoring the need for rigorous longitudinal and mechanistic studies.

#### Mechanistic evolution and biological plausibility

Mechanistic research has transformed the infectious hypothesis from a correlational concept into a biologically plausible framework. Multiple viral pathogens, including HSV-1, HIV, CMV, SARS-CoV-2, and influenza, have been shown to directly or indirectly modulate pathways central to AD, including APP processing, Aβ clearance, autophagy, and innate immune activation [25–27] A transformative line of evidence emerged from studies demonstrating that Aβ possesses antimicrobial properties, reframing amyloid deposition as a potential innate immune defense response rather than solely a pathological hallmark [7, 24, 28–30]. Viral infection has also been shown to influence Tau biology including splice variant expression, kinase activation, and phosphorylation patterns. These findings support a shift from the classical (Fig. 2) ‘infectious brain hypothesis’ toward an ‘infectious inflammatory brain hypothesis’emphasizing immune-mediated amplification of neurodegeneration.

#### Host susceptibility, genetic modifiers, and disease heterogeneity

Not all individuals exposed to neurotropic viruses develop AD, indicating significant host-specific variability. Genetic factors including APOE4, which amplifies HSV-1–driven inflammatory and amyloidogenic responses, play a central modifying role [25]. Additional contributors include age-associated immune decline, interindividual differences in innate immune signaling, frequency of viral latency and reactivation, and viral strain diversity. Accumulating evidence suggests that interactions among multiple viral exposures may shape host–pathogen dynamics, aligning with emerging polymicrobial and immune-dysregulation models of AD.

#### Therapeutic contradictions and translational gaps

Although antiviral therapies and vaccinations can reduce viral load, they have not consistently prevented or reversed cognitive decline in conditions such as HIV-associated neurocognitive disorder (HAND) or HSV-1–associated impairment [31–34]. Preclinical studies frequently demonstrate antiviral-mediated reductions in Aβ or inflammatory markers, but these findings rarely translate into clinical efficacy. Animal models pose additional limitations, including species-specific viral tropism and challenges in modeling lifelong viral latency [25]. These translational gaps suggest that once neuroinflammatory, amyloidogenic, or proteostatic cascades are activated, they may progress independently of the initial infectious trigger. Importantly, concerns that early, preclinical neurodegeneration might increase susceptibility to infection have been addressed by stratified analyses of 30-year longitudinal datasets, which support a potential causal role for infections rather than reverse causality [35].

#### Polymicrobial burden and chronic inflammatory load

Growing evidence supports an expanded infectious model involving multiple latent or persistent pathogens, such as HSV-1, CMV, EBV, and fungi, whose cumulative reactivation over the lifespan drives chronic microglial priming, inflammasome activation, and impaired proteostasis [36]. This prospective aligns with broader concepts of “inflammaging” and better accounts for mechanistic convergence across disparate pathogens, although it complicates efforts to develop single-pathogen targeted therapies.

#### Persisting gaps, conflicting data, and overinterpretation risks

Despite mounting evidence, key uncertainties remain. Critical gaps include limited long-term temporal data confirming that infection precedes AD pathology, difficulty distinguishing causative infections from opportunistic colonization, and the absence of definitive non-human primate transmission models. Conflicting studies, such as those failing to detect viral markers or showing no cognitive associations underscore the need for caution. A balanced interpretation recognizes infection as a meaningful contributor within a multifactorial landscape that includes genetic factors, metabolic dysfunction, cerebrovascular impairment, and environmental exposures. Continued integration of multi-omics cohorts, viral-host interactome mapping, and improved mechanistic study platforms (e.g., human organoids, humanized immune-competent mouse models) will be essential for clarifying causality and therapeutic relevance.

### Mechanistic links between virus specific infection and AD/ADRD

#### Rationale for virus selection

A few neurotropic and systemic viruses prioritized in this review were selected because each meets three pre-specified criteria: (i) population-level epidemiological evidence linking infection or reactivation to AD or all-cause dementia risk, (ii) mechanistic data demonstrating direct or indirect engagement of core AD pathways (Aβ processing, Tau phosphorylation, NLRP3-driven neuroinflammation, or BBB disruption), and (iii) availability of systems-biology datasets sufficient to support cross-pathogen network analyses. Several other neurotropic viruses with documented links to AD-related pathology were excluded from the main discussion and addressed in Supplementary Table 2 because they did not satisfy all three criteria, or because their mechanistic contribution is largely subsumed within the pathways already examined for those discussed in more details in our review. The current knowledge of select viral infections with potential links to neuroinflammation, neurodegeneration and AD/ADRD is provided below.

### HSV-1

HSV-1 is a highly prevalent neurotropic virus that infects over 70% of the global population and establishes lifelong latency within peripheral and central nervous system tissues [37]. Epidemiological studies, including cohort and biobank analyses, have reported associations between HSV-1 infection or recurrent herpes labialis and an increased risk of AD or ADRD [38].

**Fig. 3:**
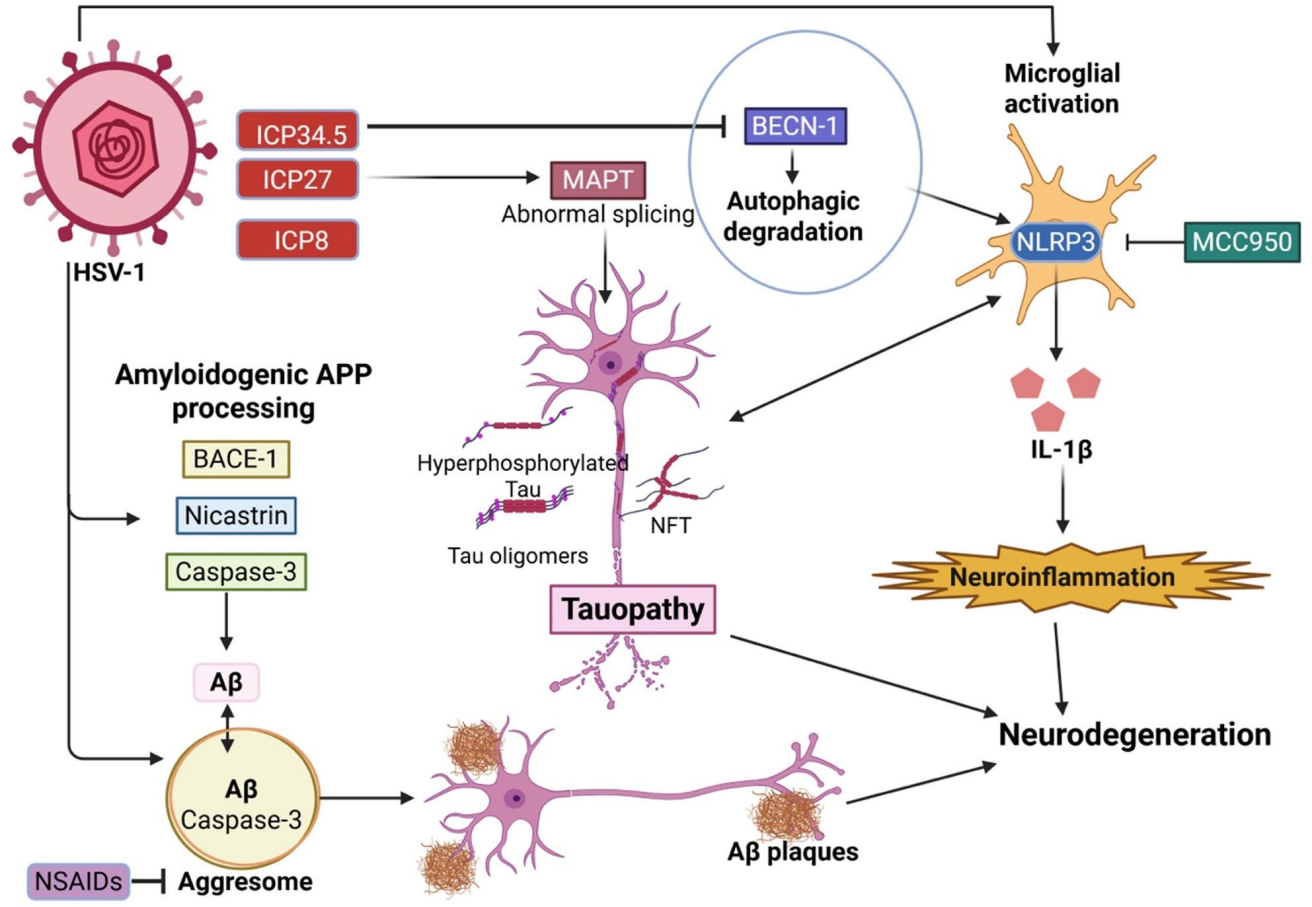
HSV-1-Induced Mechanisms Contributing to AD. HSV-1 infection promotes Alzheimer’s pathology through multiple interconnected mechanisms. Viral protein ICP27 alters Tau splicing; ICP34.5 inhibits BECN1-mediated autophagy; BACE-1, nicastrin, and caspase-3 are upregulated, increasing Aβ and aggresome formation. Concurrent activation of the NLRP3 inflammasome triggers IL-1β–mediated neuroinflammation. Microglial activation and Tauopathy form a reinforcing cycle. MCC950 inhibits NLRP3, reducing Aβ and tau pathology

However, these associations remain largely correlative and are limited by heterogeneity in diagnostic criteria and dataset coding. In contrast, mechanistic evidence derived from cellular models, human brain organoids, and animal studies provides strong biological plausibility for HSV-1-mediated contributions to AD-like pathology (Fig. 3). HSV-1 infection alters amyloid precursor protein (APP) processing by upregulating β-secretase (BACE1) and nicastrin, thereby enhancing amyloidogenic cleavage and increasing production of Aβ₁–₄₀ and Aβ₁–₄₂ [39]. Aβ₁–₄₂ preferentially accumulates due to its higher aggregation propensity [40]. In vivo studies demonstrate accelerated amyloid deposition without increases in APP expression, implicating impaired microglial clearance rather than excess Aβ production [41]. Mechanistically, HSV-1 redirects microglial phagocytosis toward infected neurons, reducing Aβ₁–₄₂ uptake and increasing plaque burden, while experimental studies have refuted the hypothesis of viral mimicry–driven amyloidogenesis [39].

Beyond amyloid pathology, HSV-1 engages multiple convergent molecular pathways relevant to AD. Viral infection activates caspase-3, which cleaves APP in a manner favoring Aβ₁–₄₂ generation [42]. Notably, Aβ₁–₄₂ and activated caspase-3 accumulate within vimentin-encased aggresomes that suppress apoptosis, allowing infected neurons to persist as chronic sources of amyloid [43, 44]. Aβ₁–₄₂ oligomers are selectively detected in cells expressing the viral replication protein ICP8, linking active viral replication to amyloid production [45]. HSV-1 also promotes tau pathology. The viral protein ICP27 disrupts MAPT splicing, shifting the 3R:4R tau ratio toward aggregation-prone 4R tau and driving tau hyperphosphorylation, neuritic degeneration, and cytoskeletal instability reminiscent of AD [46, 47]. Concurrently, HSV-1 activates the NLRP3 inflammasome, elevating IL-1β levels, suppressing PP2A activity, and enhancing GSK-3β signaling, thereby intensifying tau pathology and neuroinflammation [41, 48, 49]. Hyperphosphorylated tau further activates glial cells, establishing a self-reinforcing cycle of inflammation and neurodegeneration [50]. Additional mechanisms include ICP34.5-mediated inhibition of autophagy through BECN1 suppression [51], BBB disruption through cytokine-driven ICAM-1 expression [37], and reinforcement of feed-forward inflammatory signaling loops [52, 53]. Collectively, these findings support HSV-1 as a biologically plausible risk-modifying factor, rather than a singular causal agent, in AD pathogenesis.

#### HIV

**Fig. 4:**
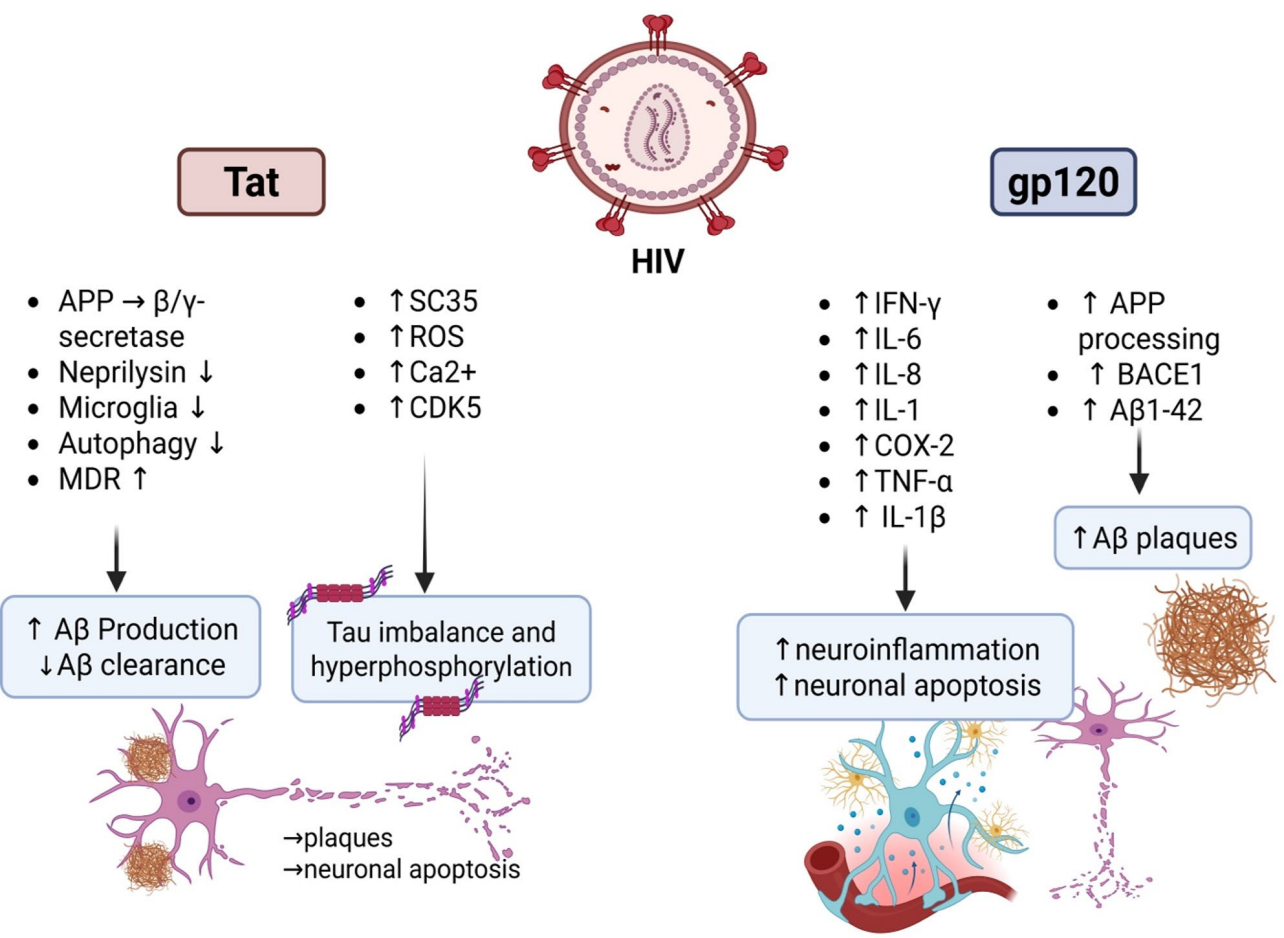
HIV-1 Tat and gp120 Proteins in AD Pathology HIV-1 Tat protein promotes amyloidogenic processing of APP via β- and γ-secretase activity and suppresses Aβ clearance via downregulation of neprilysin, microglial phagocytosis, autophagy, and blood-brain barrier efflux proteins (MDR). Tat also promotes tau hyper-phosphorylation through SC35 modulation, ROS/Ca²⁺ elevation, and CDK5 activation, leading to neuronal death. gp120 exacerbates neuroinflammation via pro-inflammatory cytokines and promotes amyloid pathology through upregulation of BACE1 and Aβ1–42 upregulation

HIV affects more than 39 million people globally and establishes long-term persistence within the nervous system. Approximately 50% of individuals with chronic HIV infection develop HAND [54], a spectrum of cognitive impairment that shares several clinical and pathological features with early AD [55]. However, unlike HSV-1, epidemiological evidence linking HIV to AD/ADRD remains limited and largely indirect, often mediated through HAND rather than AD-specific pathology. In contrast, mechanistic and experimental evidence schematically shown in Fig. 4, provides a stronger biological rationale for how chronic HIV infection, especially through viral proteins such as Tat and gp120, may lower the threshold for AD-related neurodegeneration. Tat is detectable in the CSF of HIV-positive individuals even during effective ART [56], indicating persistent biological activity. Experimental studies show that Tat enhances amyloidogenic processing by redistributing APP to compartments enriched in β- and γ-secretase activity, thereby increasing Aβ1–42 production [55]. Tat also inhibits microglial phagocytosis, disrupts lysosomal function, and suppresses STAT1-driven autophagy, collectively reducing Aβ clearance [7, 55, 57]. Additional in vitro work demonstrates that Tat promotes β-sheet formation and stabilizes aggregation-prone Aβ conformations, generating mixed Tat–Aβ fibrils that exhibit greater neurotoxicity relative to Aβ alone [58, 59].

Beyond amyloid pathways, HIV also influences Tau biology. Tat-induced activation of DYRK1A and subsequent SC35 phosphorylation alters MAPT splicing, increasing 3R-Tau isoform production and contributing to isoform imbalance associated with AD [60]. Tat uptake by neurons and astrocytes raises intracellular Ca²⁺ and reactive oxygen species (ROS), activating CDK5 and further promoting Tau hyperphosphorylation [17].Another key HIV protein, gp120, independently contributes to neuroinflammation and AD-like pathology [7, 61]. gp120 induces robust cytokine production, including IL-1β, IL-6, IFN-γ, TNF-α, enhances BACE-1 expression, increases Aβ1–42 levels, and disrupts BBB integrity [62]. Diffuse Aβ plaques described in HIV-positive individuals resemble early-stage AD plaques, although these findings remain inconsistent and may reflect HAND rather than true AD pathology [63]. In summary, HIV functions as a risk-modifying virus that exacerbates AD-related pathology through chronic neuroinflammation [61, 64], persistent viral protein activity, and accelerate CNS aging. Within the framework of the ‘infectious-inflammatory brain hypothesis’, HIV is best understood as a potentiating contributor that reduces resilience and accelerates pre-existing neurodegenerative processes rather than serving as a primary etiological agent.

#### CMV

**Fig. 5:**
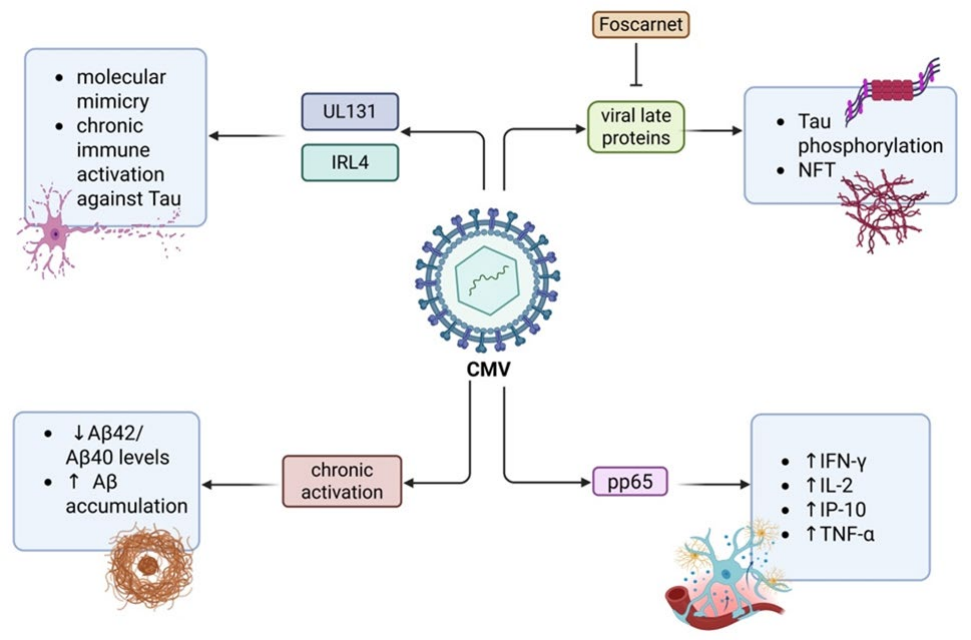
CMV-Driven Tau and Amyloid Pathology. Viral proteins UL131 and IRL4 exhibit molecular mimicry with tau protein, eliciting a T cell–mediated immune response that drives tauopathy. CMV late proteins induce tau phosphorylation and NFT accumulation (blocked by foscarnet). Reactivation (pp65 antigen) stimulates cytokines (IFN-γ, IL-2, IP-10, TNF-α), amplifying neuroinflammation. CMV also lowers Aβ42/Aβ40 ratio and increases Aβ accumulation—initially protective, but harmful with chronic activation

Cytomegalovirus (CMV) is a highly prevalent, lifelong latent herpesvirus and a major cause of congenital infection with more than half of U.S. adults infected by age 40 [65, 66]. Epidemiological links between CMV to AD or ADRD are mixed but suggestive, with some cohort studies reporting an ~ 2.4-fold increased risk of AD among CMV-seropositive individuals [67], but these findings are inconsistently replicated and often vary based on population characteristics, serological criteria, and coexisting inflammatory conditions. CMV’s relationship to AD remains correlation-level, complicated by its high prevalence, broad age distribution, and frequent subclinical reactivation. Mechanistic and experimental data, however, provide more robust support for CMV as a modulator of AD-relevant pathways. CMV infects neural progenitor cells, neurons, astrocytes, and glia, cell types central to neurodegenerative cascades. One proposed mechanism involves molecular mimicry (Fig. 5), in which CMV proteins such as UL131 and IRL4 share structural motifs with Tau [68] potentially promoting chronic immune activation and early Tau-related pathology. Experimental studies demonstrate that CMV exposure can induce high-molecular-weight Tau species and phosphorylation at serine 396 (S396), a key site implicated in neurofibrillary tangle (NFT) formation [69, 70]. These CMV-induced Tau alterations resemble changes observed in AD cerebrospinal fluid.

The evidence that inhibiting viral DNA polymerase with foscarnet blocks Tau phosphorylation and abnormal isoform shifts, indicates that active viral gene expression, rather than passive immunological imprinting, is necessary for CMV-related Tau pathology [69]. This contrasts with HIV, where stable viral proteins (Tat, gp120) drive pathology even under ART; and with HSV-1, where both latent and reactivated states influence APP processing and Tau biology. With respect to Aβ, observational studies report that CMV IgG seropositivity correlates with decreased Aβ42/Aβ40 ratios and increased amyloid deposition in experimental systems [71]. This has prompted the interpretations that Aβ production may initially function as an antimicrobial response against CMV, consistent with the infectious brain hypothesis. Chronic reactivation, however, may shift this protective response into pathological amyloidosis. CMV also elicits a strong inflammatory response, particularly during reactivation events marked by the pp65 antigen. pp65-responsive CD4⁺ T cells secrete interferon-γ (IFN-γ), a cytokine repeatedly shown to promote Tau hyperphosphorylation in animal and cellular models [72]. CMV-stimulated immune responses also secrete additional inflammatory mediators such as IL-2, IP-10, TNF-α, and ROS, collectively amplifying microglial activation and oxidative stress [73]. TNF-α and ROS upregulate MMP-9, which disrupts tight junction proteins and compromising BBB integrity, a feature shared with HIV and SARS-CoV-2, although less pronounced than in HSV-1 encephalitic models [74]. Overall, CMV does not exert the same direct mechanistic impact on canonical AD pathways as HSV-1, nor does it display the protein-specific neurotoxicity characteristic of HIV’s Tat and gp120. Instead, its combination of ubiquity, immune-reactivation cycles, and propensity to induce Tau-directed immune responses suggests that CMV functions primarily as a chronic immune amplifier that increases vulnerability to AD-related pathology, particularly in aging populations.

#### COVID-19

**Fig. 6:**
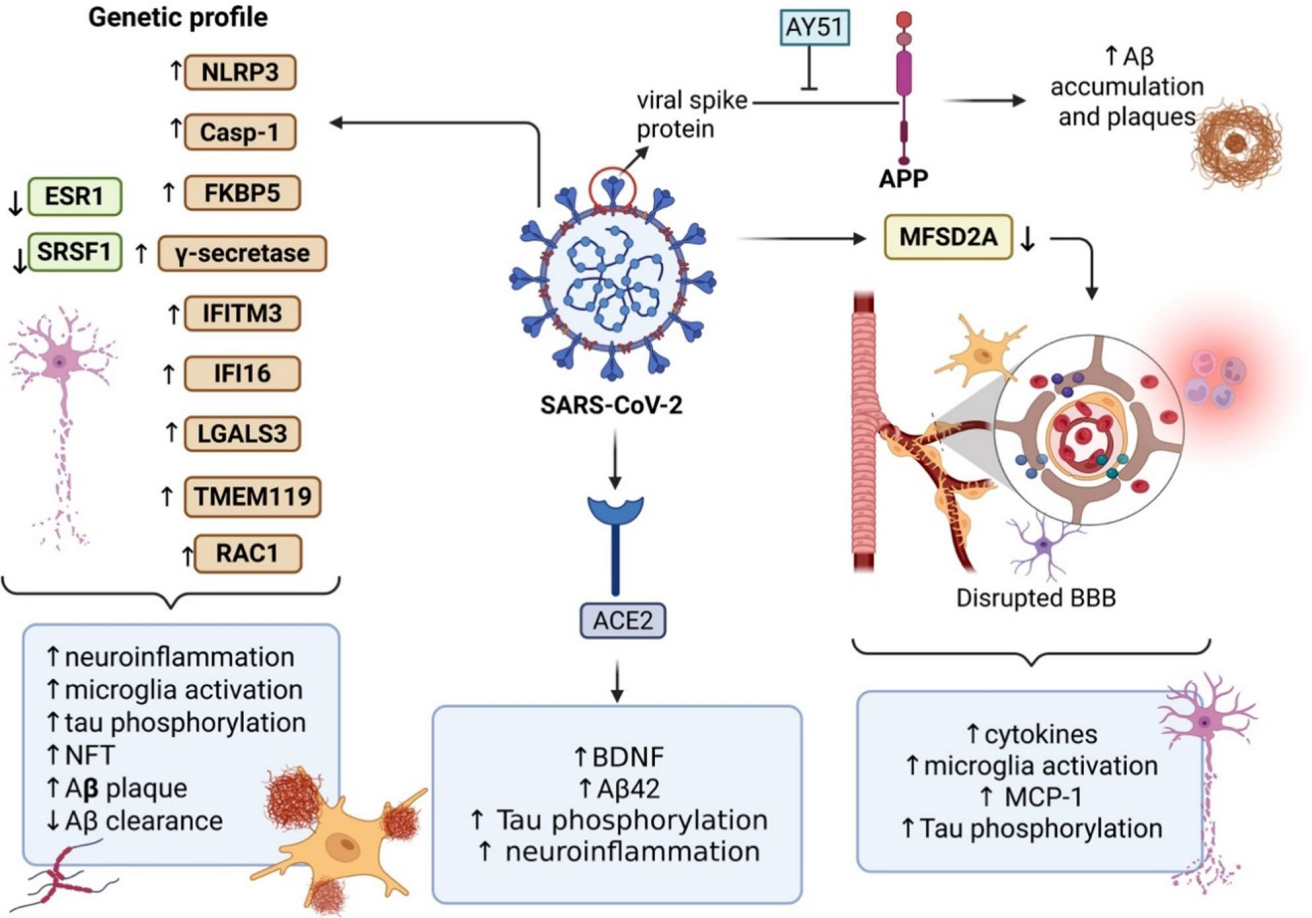
SARS-CoV-2 Mechanisms Overlapping with AD Pathology. SARS-CoV-2 infections alters gene expression by upregulating AD-related genes (NLRP3, FKBP5, IFITM3, γ-secretase) and downregulating protective genes (ESR1, SRSF1). The virus enters cells via ACE2, reducing BDNF and increasing tau phosphorylation and neuroinflammation. Its spike protein binds to APP, increasing Aβ production and plaque formation (blocked by AY51). SARS-CoV-2 also downregulates MFSD2A, disrupting the blood-brain barrier (BBB), allowing peripheral cytokines to penetrate the CNS. This promotes microglial activation, tau pathology, and neuronal injury, closely mimicking AD molecular profiles

Since emerging in 2019, SARS-CoV-2 has infected billions worldwide, raising concern regarding its long-term neurological sequelae, including possible interactions with AD and other dementias. Epidemiological evidence linking COVID-19 to increased AD/ADRD risk is growing but remains evolving and not yet definitive [75]. Large international analyses (e.g., > 3,500 adults across eight countries) suggest that long COVID is associated with elevated dementia risk, with age, disease severity, and anosmia emerging as key modifiers. However, unlike HSV-1 where strong replicated biobank-level associations exist, the epidemiological links between SARS-CoV-2 and AD remain suggestive rather than conclusive due to limited follow-up, diagnostic variability, and the recent onset of the pandemic. In parallel, mechanistic and experimental evidence is substantial and rapidly expanding (Fig. 6) providing biologically plausible pathways through which SARS-CoV-2 may accelerate or unmask AD-related processes [76]. The virus can infect or dysregulate astrocytes, microglia, and endothelial cells, impairing metabolic support and reducing neuronal viability [77]. Transcriptomic profiling has identified genes (e.g., FKBP5, LGALS3, IFITM3, and IFI16) that regulate γ-secretase activity, Tau phosphorylation, APP processing, and innate immune signaling [78]. A central mechanism involves SARS-CoV-2’s interaction with ACE2, a receptor with neuroprotective functions including reducing Aβ accumulation [36] attenuating Tau phosphorylation, and suppressing inflammation. Viral binding downregulates or functionally inhibits ACE2, decreases BDNF levels, and facilitates Tau-related neurodegeneration. SARS-CoV-2 infection also alters Tau splicing regulators such as SRSF1, favoring pro-tauopathic isoform ratios (3R/4R imbalance) [79].

Further, elevated RAC1 and ARRB1 expression in AD+ COVID brain tissue suggests increased Aβ production, while APP has been shown to bind the SARS-CoV-2 spike protein, promoting viral entry and enhancing amyloid accumulation [79]. Experimental studies demonstrate that blocking spike–APP interactions reduces viral load and amyloid pathology, suggesting a possible causal molecular interaction [80]. Moreover, the spike protein contains a heparin-binding domain that binds multiple amyloidogenic proteins, showing highest affinity for Aβ1–42, thereby accelerating peptide aggregation [81]. A third major pathway involves neuroinflammation, by SARS-CoV-2 induced cytokine activation (IL-1β, IL-2, IL-6, IL-18, TNF-α, IFN-γ, MCP-1), generating oxidative stress and dysregulating calcium signaling, both of which are known to drive Tau hyperphosphorylation [79, 82–84]. SARS-CoV-2 also activates NLRP3 inflammasome and caspase-1, promoting neurofibrillary tangle formation and impairing Aβ clearance [85, 86]. The spike S2 subunit further increases microglial activation markers (TMEM119), excitatory neuron stress markers (NMDAR2), and neuronal nitric oxide synthase (nNOS), reproducing AD-like patterns of neuroimmune dysfunction [75, 87].

Additionally, experimental and post-mortem studies demonstrate BBB disruption following SARS-CoV-2 infection. Reduced expression of MFSD2A, a transporter critical for BBB stability and a known AD biomarker, has been observed in COVID-19 brain tissue [88, 89] Post-acute neuroinflammation may result from both direct viral neuroinvasion and systemic cytokine “spillover,” priming microglia and propagating Tau pathology [75]. Collectively, SARS-CoV-2 demonstrates strong mechanistic plausibility as an accelerator of AD-related neurodegeneration, on par with, and in some pathways exceeding, HIV and CMV. However, unlike HSV-1, SARS-CoV-2 lacks decades-long longitudinal data or replicated large-scale associations definitively linking infection to AD incidence. Thus, SARS-CoV-2 should currently be viewed as a potent neuroinflammatory and neurodegenerative amplifier that may lower the threshold for AD-like pathology, particularly in individuals with preexisting vulnerabilities, while long-term epidemiological confirmation continues to accumulate.

#### Influenza

**Fig. 7:**
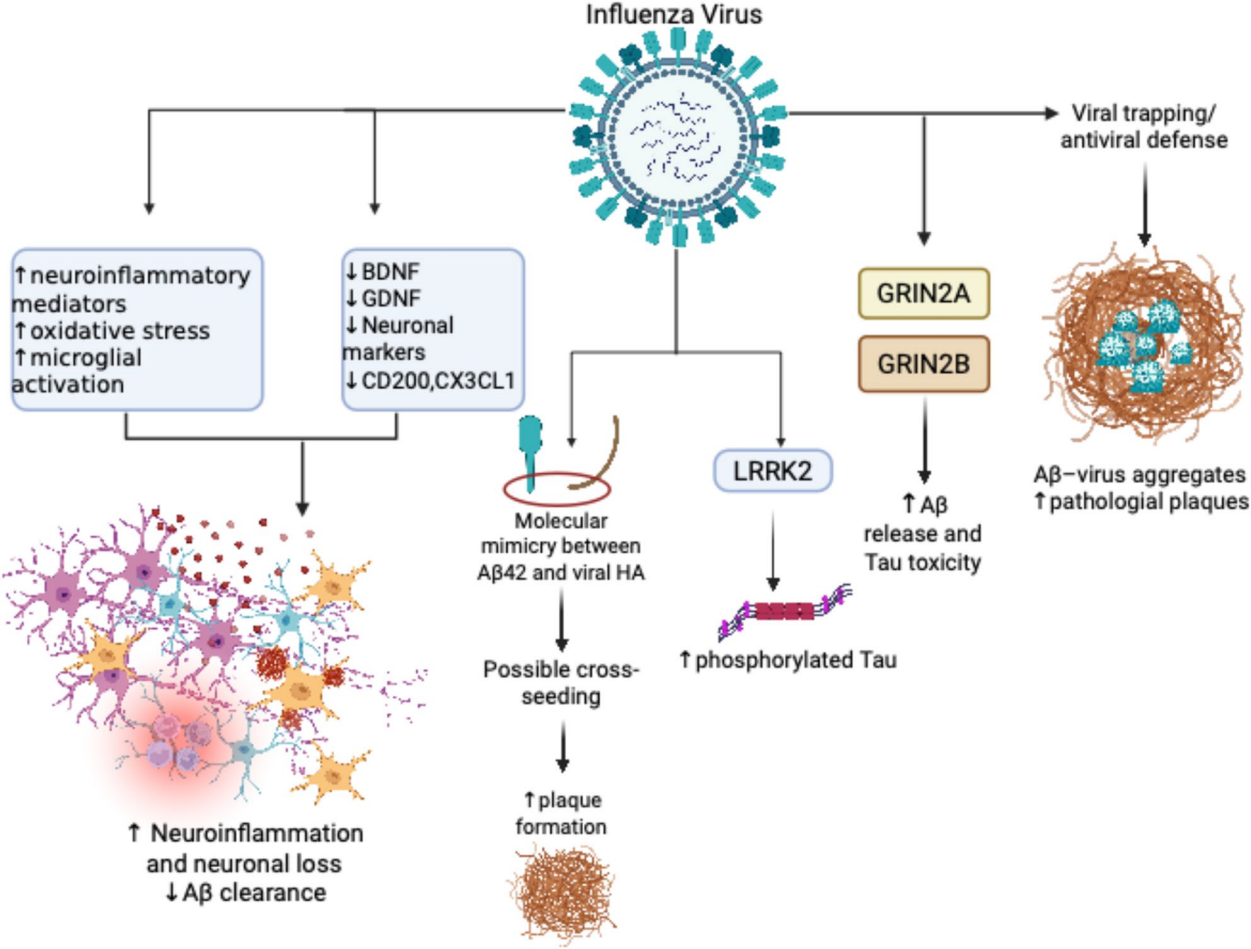
Influenza-Associated Mechanisms relevant to AD. Influenza upregulates LRRK2, increasing tau phosphorylation. The virus exhibits molecular mimicry between its hemagglutinin (HA) protein and Aβ42, leading to cross-seeding events that promote Aβ-virus aggregates and accelerate pathological plaque formation. This viral trapping may represent an antiviral defense mechanism that inadvertently increases amyloid burden. The infection upregulates NMDA receptor subunits (GRIN2A, GRIN2B), which enhances Aβ release and exacerbates tau toxicity. Concurrently, influenza triggers neuroinflammation by elevating pro-inflammatory mediators and oxidative stress while activating microglia

The epidemiological evidence linking influenza infection [90] to AD or ADRD is comparatively weaker and less consistent compared with herpesviruses (HSV-1, CMV) and SARS-CoV-2. Mechanistic and experimental research, however, provides biologically plausible pathways through which influenza infection can contribute to AD-relevant neurodegeneration (Fig. 7). Certain avian strains such as Influenza Strain H5N1 may also influence Tau biology, albeit most seasonal influenza viruses do not directly infect the CNS. Specifically, H1N1 infection has been linked to LRRK2-dependent Tau phosphorylation, which is reversible with LRRK2 inhibitors in animal models [91]. Upregulation of GRIN2A/GRIN2B (encoding NMDAR subunits) promotes Aβ release and contributes to Tau-dependent toxicity through GSK-3β signaling. Influenza A also activates the IRE1-regulated unfolded protein response (UPR), causing chronic ER stress, which is an established driver of misfolded protein accumulation in AD.

Beyond amyloid and Tau pathways, influenza affects neuronal resilience and immune homeostasis. Infection reduces levels of BDNF and GDNF, both critical for neuronal survival and synaptic plasticity, while downregulating CD200 and CX3CL1, key immunoregulatory molecules controlling microglial reactivity [91, 91]. These shifts create a microenvironment favoring chronic inflammation and impaired neuronal repair. Additionally, influenza’s neuraminidase-induced desialylation may also influence AD-related responses, given the importance of sialic acid in synaptic stability and Siglec-mediated immune signaling [90, 92]. Altered sialic acid metabolism, also observed in AD brains, raises the possibility that influenza-related desialylation could disrupt interactions involving Siglec-3 (CD33), a recognized AD risk gene [93, 94].

Thus, influenza demonstrates moderate mechanistic plausibility as a contributor to AD-relevant neuroinflammation, primarily through cytokine activation, microglial priming, and stress-response pathways affecting Aβ and Tau. However, epidemiological evidence linking influenza infection to AD remains weaker than for HSV-1 or SARS-CoV-2, and direct causality has not been established. Instead, influenza appears to act as a systemic inflammatory amplifier capable of exacerbating neurodegenerative processes in susceptible individuals. By contrast, influenza vaccination shows consistent protective associations, highlighting the central role of inflammation rather than direct neurotropism as the principal driver of influenza-related cognitive risk [95]. Within the framework of the Infectious Brain Hypothesis, influenza should therefore be considered a secondary, inflammation-mediated contributor to AD risk rather than a direct neurotropic driver of AD pathology.

#### Epstein–barr virus (EBV)

Epstein–Barr virus (EBV), a γ-herpesvirus establishing lifelong latency in B lymphocytes, is increasingly implicated in AD/ADRD through immune-mediated and inflammatory mechanisms rather than direct neuronal infection. EBV reactivation induces chronic peripheral immune activation, elevating pro-inflammatory cytokines such as IFN-γ, TNF-α, and IL-6 that can traverse a compromised blood–brain barrier (BBB) and promote microglial priming and astrocytic activation [96, 97]. EBV-infected B cells may infiltrate the CNS and serve as antigen-presenting cells, sustaining long-term innate immune activation. At a molecular level, EBV-driven immune dysregulation and epigenetic remodeling amplify NF-κB and interferon signaling, increasing oxidative stress, impairing amyloid-β (Aβ) clearance, and activating Tau-phosphorylating kinases. These pathways converge on AD-relevant processes, positioning EBV as a chronic immune amplifier that lowers the threshold for neurodegeneration, particularly in aging or genetically susceptible individuals [96, 97].

#### Zika virus (ZIKV)

Zika virus (ZIKV), a neurotropic flavivirus, demonstrates strong mechanistic links to AD-like pathology through direct infection of neurons, neural progenitor cells, astrocytes, and microglia. ZIKV activates innate immune pathways (TLR3, RIG-I, cGAS–STING), triggering sustained type I interferon responses and chronic neuroinflammation [98, 99]. ZIKV infection induces endoplasmic reticulum stress and the unfolded protein response, resulting in PERK-eIF2α activation and downstream stimulation of BACE1 and GSK-3α/β, leading to increased Aβ production and Tau hyperphosphorylation [100].Concurrent disruption of autophagic–lysosomal pathways impairs Aβ clearance, while BBB breakdown facilitates immune cell infiltration. These combined cellular insults recapitulate key AD hallmarks, indicating that ZIKV infection may prime or accelerate neurodegenerative cascades when inflammatory resolution is incomplete.

#### Varicella–zoster virus (VZV; VLV)

Varicella–zoster virus (VZV), an α-herpesvirus capable of latency and reactivation, is mechanistically linked to AD/ADRD through vascular inflammation and BBB dysfunction. Reactivated VZV can infect cerebral arteries and endothelial cells, causing vasculitis, microinfarction, and chronic endothelial activation. Cellular consequences include upregulation of ICAM-1 and VCAM-1, activation of matrix metalloproteinases (MMP-2/9), and degradation of tight junction proteins, resulting in BBB leakage [101, 102]. Molecularly, VZV-induced NF-κB signaling, oxidative stress, and cytokine release promote amyloidogenic APP processing and Tau-phosphorylating kinase activation. Large population-level studies further associate VZV reactivation with increased dementia risk, while herpes zoster vaccination reduces this risk, underscoring the relevance of VZV-driven inflammatory injury in neurodegenerative trajectories.

#### Human herpesvirus-6 (HHV-6)

Human herpesvirus-6 (HHV-6), particularly HHV-6 A, is a highly neurotropic β-herpesvirus capable of infecting neurons, astrocytes, oligodendrocytes, and microglia, and is uniquely able to integrate into the human genome. HHV-6 infection alters host transcriptional and epigenetic programs governing APP metabolism, synaptic signaling, and innate immune activation [103, 104]. Cellular studies show that HHV-6 promotes microglial activation and astrocytic dysfunction, leading to persistent neuroinflammation. At a molecular level, HHV-6 A viral proteins increase APP expression, inhibit its degradation, enhance Aβ accumulation, and activate MAPK and NF-κB signaling pathways that favor Tau phosphorylation. Meta-analyses of brain tissue and PCR-based studies demonstrate a significant association between HHV-6 infection and increased AD risk, supporting its role as a latent, reactivation-driven contributor to proteostatic failure and neurodegeneration.

#### Human herpesvirus-7 (HHV-7)

Human herpesvirus-7 (HHV-7), a β-herpesvirus closely related to HHV-6, has more limited direct mechanistic data but is increasingly recognized as a contributor to cumulative neuroinflammatory load in AD/ADRD. HHV-7 primarily infects CD4⁺ T cells but has been detected in CNS tissue, suggesting indirect effects mediated through chronic immune activation. Recurrent HHV-7 reactivation perpetuates cytokine and interferon signaling, oxidative stress, and NF-κB activation, all of which intersect with pathways regulating Aβ aggregation, Tau phosphorylation, and synaptic dysfunction [96, 97]. Within a viral exposome framework, HHV-7 likely acts synergistically with other herpesviruses to sustain low-grade inflammation and microglial priming, accelerating downstream neurodegenerative processes rather than acting as a solitary etiologic agent.

Collectively, these viral infections converge on a shared cellular–molecular architecture characterized by innate immune activation, BBB disruption, impaired autophagy–lysosomal function, and chronic neuroinflammation, despite differences in tropism and life cycle. These infections amplify Aβ dysregulation, Tau pathology, vascular injury, and synaptic loss, functioning predominantly as risk modifiers and accelerants that lower resilience to neurodegeneration in aging or genetically susceptible individuals. This convergence strongly supports infectious-exposome and polymicrobial models of AD/ADRD pathogenesis rather than single-agent causality.

### Cross-virus comparison

Table 1 summarizes the epidemiologic and mechanistic evidence across major neurotropic and systemic viruses, together with a critical evaluation of their evidentiary strength. Across HSV-1, HIV, CMV, SARS-CoV-2, and influenza, there is substantial heterogeneity in both the strength of epidemiological associations and the depth of mechanistic evidence linking individual viruses to AD. HSV-1 exhibits the strongest and most consistent epidemiological signal, particularly among APOE4 carriers, and is supported by extensive mechanistic data demonstrating direct effects on APP processing, Aβ deposition, Tau dysregulation, and inflammasome activation. SARS-CoV-2 shows rapidly emerging but still maturing epidemiological evidence, while mechanistic studies have already identified multiple convergent pathways including ACE2 inhibition, spike–APP binding, NLRP3 activation, and BBB disruption that plausibly accelerate AD-related neurodegeneration. HIV demonstrates strong mechanistic evidence, primarily through Tat and gp120-mediated Aβ and Tau alterations, yet display weak and inconsistent epidemiological links, suggesting its cognitive impact is driven principally by HAND-related neuroinflammation rather than AD-specific processes. CMV presents mixed epidemiological associations and moderate mechanistic support centered on immune-mediated Tau mimicry, chronic inflammation, and BBB compromise, positioning it as an indirect risk amplifier rather than a primary driver of AD. Influenza shows the weakest epidemiological association with AD, with most evidence indicating that systemic inflammation, not direct neurotropism, as its primary contribution to neurodegeneration; notably, influenza vaccination shows more consistent protective associations than infection shows risk. Collectively, these data highlight that while multiple viruses converge on shared inflammatory and proteostatic pathways, only a subset, most notably HSV-1 and potentially SARS-CoV-2, demonstrate strong integration across epidemiologic and mechanistic evidence, while others exert more indirect, context-dependent, or host-mediated effects within the broader Infectious Brain Hypothesis.

**Table 1:** Evidence Linking Major Viruses to AD/ADRD

Virus	Epidemiological Evidence Linking to AD/ADRD	Mechanistic / Experimental Evidence	Relative Strength of Evidence	Overall Interpretation
HSV-1	Strongest epidemiological signal; multiple replicated biobank studies show increased AD risk, especially in APOE4 carriers. Associations persist across cohorts and decades.	Extensive mechanistic evidence: direct manipulation of APP processing; ↑Aβ1–40/42; impaired microglial clearance; Tau splicing changes (ICP27); inflammasome activation (NLRP3); BBB disruption (ICAM-1, cytokines).	Highest among reviewed viruses due to consistent human associations + clear mechanistic pathways.	Likely a major risk-modifying pathogen with plausible direct contribution to Aβ and Tau pathology.
HIV	Weak for AD specifically; HAND is common, but epidemiological studies do not show consistent increased AD incidence. ART survival patterns complicate analysis.	Strong mechanistic evidence, largely via viral proteins (Tat, gp120): ↑Aβ production; ↓Aβ clearance; Tau splicing imbalance; oxidative stress; NLRP3 activation; neuronal apoptosis; BBB disruption.	Moderate (high mechanistic, low epidemiologic).	HIV acts as a risk-lowering-resilience factor via chronic inflammation and viral protein toxicity—not a direct AD cause.
CMV	Mixed associations; some cohorts show ~ 2.4× increased AD risk, others show no association. High prevalence complicates signal detection.	Moderate mechanistic support: Tau molecular mimicry (UL131, IRL4); Tau S396 phosphorylation; immune-driven inflammation (IFN-γ, TNF-α); ↓Aβ42/40 ratios; MMP-9–mediated BBB disruption.	Moderate, driven more by immune effects than direct neuronal mechanisms.	CMV likely acts as a chronic inflammatory amplifier, promoting Tau pathology through immune cross-reactivity.
SARS-CoV-2	Rapidly emerging evidence: long COVID cohorts show increased dementia diagnoses; still early and lacking long-term follow-up. Not yet conclusive.	Strong mechanistic evidence: ACE2 inhibition → ↑Tau phosphorylation; spike–APP binding → ↑Aβ; inflammasome activation (NLRP3); massive cytokine surge; BBB disruption (↓MFSD2A); oxidative stress; persistent microglial activation.	High mechanistic, moderate epidemiologic; second strongest after HSV-1.	SARS-CoV-2 is a potent accelerator of AD-like pathways, likely lowers neurodegenerative threshold, but long-term causal links still emerging.
Influenza	Weak, inconsistent associations; infection ≠ proven AD risk. Stronger evidence for AD-risk reduction with vaccination than for harm from infection.	Moderate mechanistic support: systemic inflammation → microglial priming; ↑Aβ deposition; Tau phosphorylation (LRRK2-dependent); IRE1/UPR activation; ↓BDNF/GDNF; Siglec/CD33 modulation via desialylation.	Moderate-to-low, primarily indirect via inflammation rather than direct CNS effects.	Influenza acts as an inflammatory stressor, potentially exacerbating neurodegeneration in susceptible individuals; vaccination appears protective.

### Potential for convergence in virus and brain interactions

The public health implications of these mechanistic convergences are substantial. Approximately 80–90% of adults acquire HSV-1 by late middle age, 40 million people worldwide live with HIV, and CMV infection is similarly widespread [105, 106]. Most consequentially, the unprecedented global reach of the SARS-CoV-2 raises concerns that convergent viral mechanism may produce a future surge in neurodegenerative disease, especially among individuals with persistent post-acute neurological symptoms [107].

However, this mechanistic plausibility must be balanced against epidemiological and translational limitations before causal inferences can be drawn. Importantly, the infectious hypothesis should not be viewed in isolation but rather as one component interacting with the multifactorial etiology of AD [108]. For example, most HSV-1 carriers do not develop AD, indicating that infection is insufficient for pathogenesis [109, 110]. A major limitation in current literature is inadequate adjustment for APOE genotype, the strongest known genetic risk factor for late-onset AD. APOE4 carriers have up to a 15-fold increased risk of AD, making APOE genotype a potential confounder and a modifier of pathogen-AD association [108, 111]. Studies that do not rigorously account for APOE4 may therefore struggle to differentiate whether viral infections act as independent causal exposures, or reflect increased vulnerability in genetically susceptible individuals. Thus, the infectious hypothesis must be integrated within the broader, multifactorial landscape of AD risk.

### Cellular and molecular convergence

**Fig. 8:**
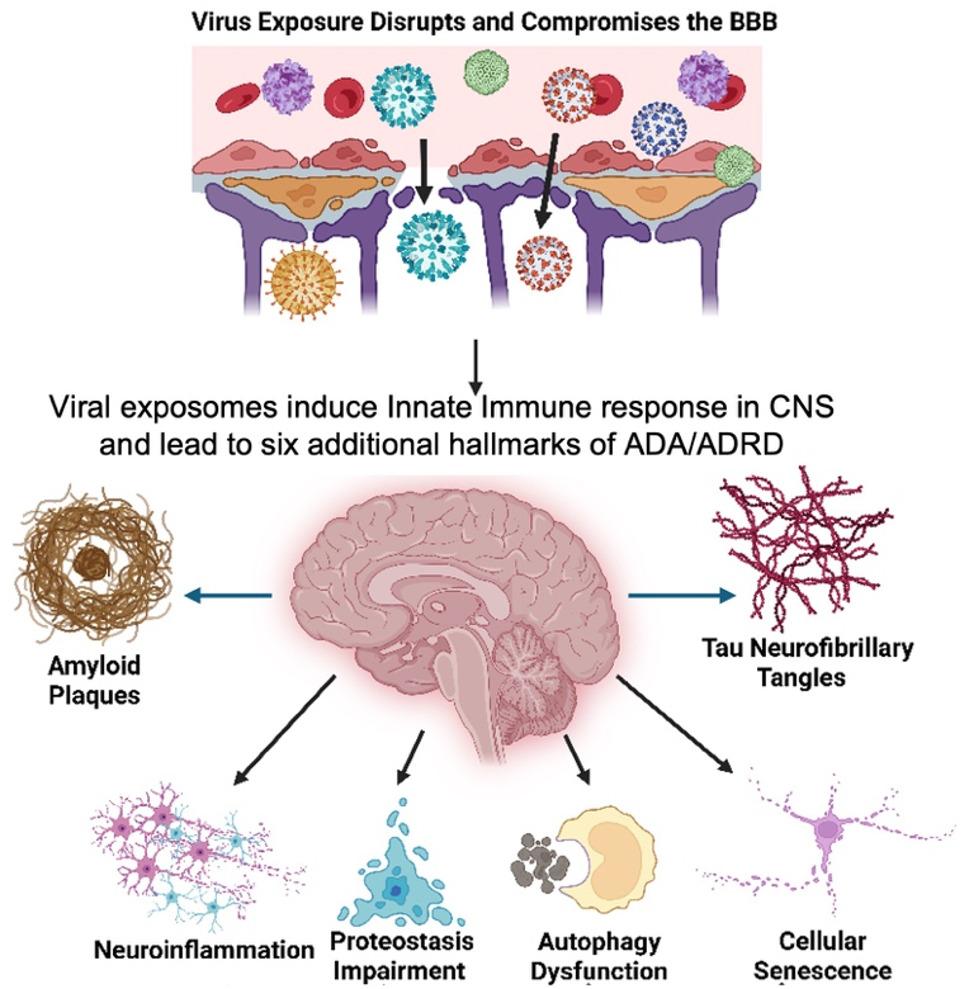
The viral exposomes converge on activation of cellular processes linked to the hallmarks of AD and ADRD. Acute infection disrupts the blood brain barrier resulting in exposure of the CNS to atypical host and viral factors which promote the maintenance of neuroinflammation. Persistent dysregulation of the BBB along with innate immune activation leads to chronic low-grade neuroinflammation, which in turn promotes glial cell dysfunction impairing their protein homeostasis and phagocytic functions. Glial cells take on a senescent phenotype in which they secrete pro-inflammatory cytokines and become deficient in their ability to clear cell debris and misfolded proteins thus promoting plaque deposition and Tau aggregation

AD arises from complex interactions between genetic susceptibility and environmental factors. Variability in findings across studies reflects the complex interactions between viral infection and other contributors to AD. This heterogeneity underscores the need to shift from one-size-fits-all models toward biomarker-guided frameworks that account for disease risk factors, epidemiological context, and translational constrains [112, 113]. Molecular convergence between neurotropic viral infection and AD pathobiology involves overlapping mechanisms that accelerate hallmark AD processes like Aβ accumulation, tau tangles, and chronic neuroinflammation (Fig. 8**)**. HSV-1, HHV-6, HHV-7, and other viruses can trigger or amplify processes already implicated in AD, particularly in genetically susceptible individuals. Several mechanisms for the cellular and molecular convergence exist, and they are not mutually exclusive [113, 114]. Lifelong, recurrent exposure to neurotropic viruses (e.g., HSV-1, CMV, HIV, SARS-CoV-2, influenza) produces synergistic, rather than merely additive, effects on brain health [115, 116]. Distinct viral mechanisms ultimately converge onto shared inflammatory and proteostatic pathways that amplify AD pathology, particularly among APOE4 carriers [96, 107]. We organize the cellular and molecular convergence between neurotropic viral infections and AD pathobiology into four hierarchical tiers: (1) First-order convergence, defined by shared innate immune activation, including microglial priming, NLRP3 inflammasome signaling, and pro-inflammatory cytokine release, (2) Second-order convergence, characterized by amplification of the two core AD proteinopathies; Aβ accumulation and Tau hyperphosphorylation, (3) Third-order convergence, reflecting failure of essential cellular homeostatic systems, including autophagy, lysosomal degradation, and the BBB, and (4) Fourth-order convergence, encompassing network-level neurodegeneration manifested as synaptic loss, circuit dysfunction, and accelerated cognitive decline.

#### First order convergence: innate immune activation

At the initial level of synergy, diverse neurotropic viruses including HSV-1, CMV, HIV, SARS-CoV-2, and influenza activate overlapping innate immune pathways, despite their distinct biology. A shared capacity to trigger microglial activation, NLRP3 inflammasome signaling, and pro-inflammatory cytokine release (IL-1β, IFN-γ, TNF-α) creates a common neuroinflammatory landscape [113, 114]. Each viral exposure or latent reactivation acts as an inflammatory “hit,” lowering microglial activation thresholds and converting acute responses into chronic, self-sustaining inflammation. This first-order convergence forms the upstream foundation upon which downstream neurodegenerative pathology unfolds [96].

#### Second order convergence: amyloid and tau pathology amplification

At the second level, viruses converge on the two core AD proteinopathies: Aβ accumulation and Tau hyperphosphorylation. HSV-1 increases Aβ production via BACE1 upregulation; CMV promotes Tau S396 phosphorylation; HIV Tat increases Aβ42 accumulation; SARS-CoV-2 affects APP processing and Tau phosphorylation; and influenza enhances Aβ release and Tau phosphorylation [96, 113]. Together, these mechanisms establish a reinforcing cycle: Aβ accumulation increases Tau pathology, and Tau alterations amplify neuroinflammation driving progression of AD, particularly in individuals with repeated viral exposures [107].

#### Third order convergence: failure of core cellular systems

At the third level, repeated viral insults degrade fundamental neuronal homeostatic systems. Chronic inflammation and viral proteins impair autophagy, disrupt lysosomal degradation, and compromise the BBB through tight-junction loss and reduced MFSD2A transporter integrity [114]. Microglia become dysfunctional, losing debris-clearance efficiency and adopting neurotoxic phenotypes [96, 113]. These combined failures mark the progressive collapse of neuronal protective networks and heighten susceptibility to neurodegeneration.

#### Fourth order convergence: network-level neurodegeneration

At the highest level of convergence, accumulated immune dysregulation, proteinopathy, and cellular failure manifest as widespread neuronal network damage consistent with clinical AD. These processes ultimately lead to widespread synaptic loss, network dysfunction, and accelerated cognitive decline [107]. This emergent phenotype reflects the cumulative burden of lifelong viral exposures, with accelerated cognitive decline most pronounced in APOE4 carriers [96, 113].

Multiple neurotropic viruses (e.g., HSV-1, HIV, CMV, SARS-CoV-2, influenza) activate overlapping host responses associated with Aβ dysregulation, Tau hyperphosphorylation, chronic neuroinflammation, and network degeneration, suggesting a shared molecular architecture across distinct pathogens [27, 117, 118]. Mechanistically, infections can increase amyloidogenic APP processing and reduce Aβ clearance; in parallel, Aβ itself exhibits antimicrobial properties, reframing amyloidosis as a potential innate defense mechanism that becomes maladaptive under persistent exposure [117, 118]. Viral activity can also modulate Tau biology, with HSV-1 and SARS-CoV-2 linked to Tau phosphorylation/aggregation and altered splicing or proteostasis, further coupling proteopathy to inflammation [25, 119–121]. These convergent pathways are amplified in APOE4 carriers, underscoring gene-environment interactions [27].

**Fig. 9:**
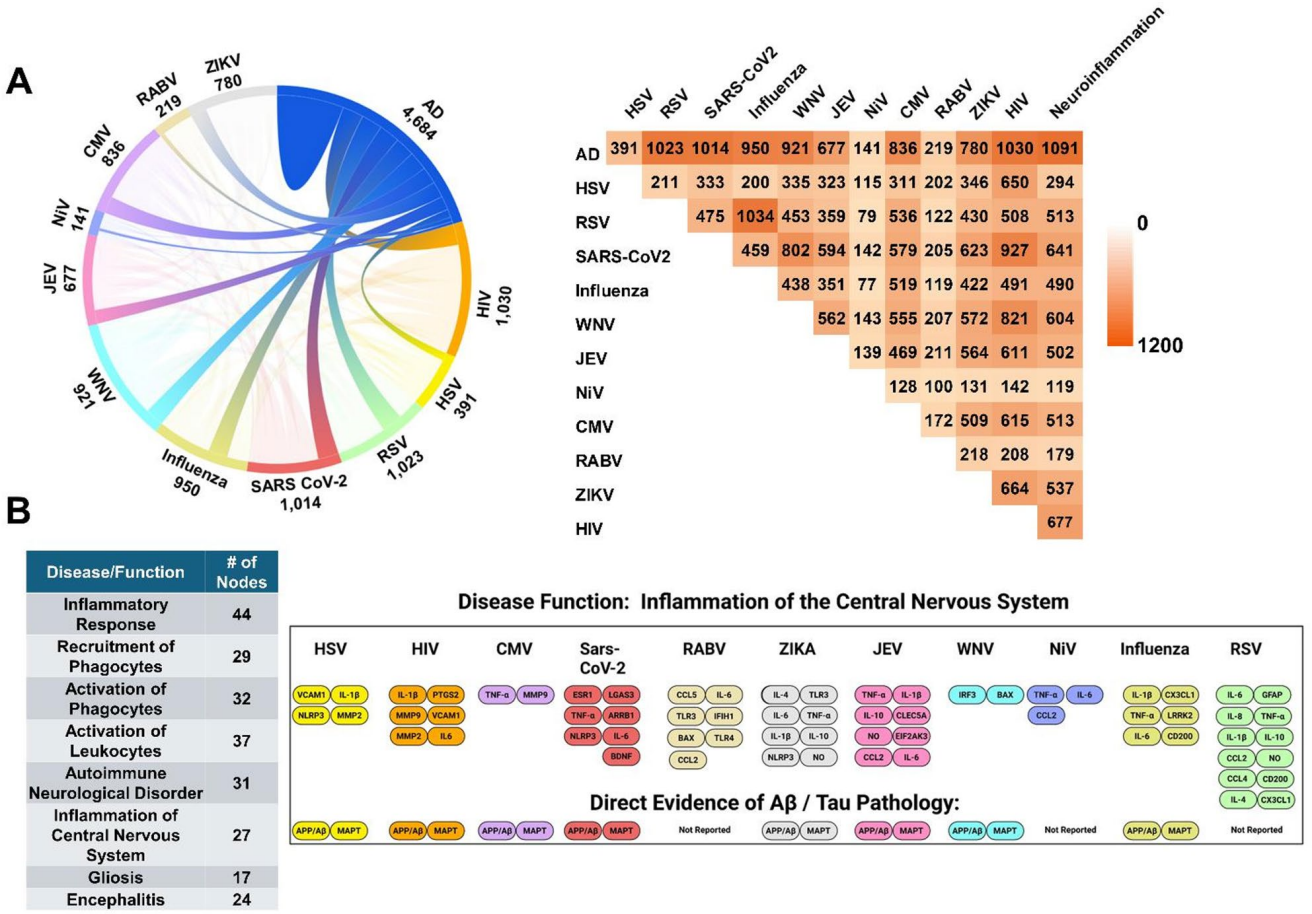
Host responses to viral infection overlap with the biological processes of AD and converge on inflammation of the central nervous system. Gene ontology terms associated with disease processes of AD, viral infections, and neuroinflammation were obtained from the STRING: Diseases database and filtered for the GO types “biological process” and “molecular function”. GO terms associated with each virus were compared to the GO terms associated with AD. The number of GO terms shared between AD and each virus are shown in a chord diagram and table (**A**). Pathway analysis was performed on the network of over 80 viral response genes and cellular functions (nodes) outlined in Supplementary Table 2 using Ingenuity Pathway Analysis software (Qiagen). Enriched disease function associations were identified using the overlay feature to display disease processes in which nodes within the network are known to participate. Disease functions over-represented in the network were ranked by p value (*p* ≤ 0.05, Fisher’s Exact Test) and the nodes contributing to the “inflammation of the central nervous system” disease function are shown for each virus (**B**)

At a system level, these molecular disturbances cascade into circuit dysfunction, synaptic loss, dendritic retraction, mitochondrial stress, mapping onto cognitive domains affected in AD/ADRD. Large biobank studies further associate diverse viral exposures (including viral encephalitis and influenza with pneumonia) with elevated long-term AD risk, consistent with a lifetime “viral exposome” model that accelerates neurodegenerative trajectories [21]. Bioinformatic and systems analyses further reveal virus–host interactomes and AD pathways (e.g., neuroinflammation, cytokine storm, MAPK/NF-κB signaling), enhancing hypothesis generation for biomarkers and interventions [113]. The literature-driven mapping of infection-associated proteins revealed numerous molecular targets, which informed Gene Ontology (GO) analyses using pathogen-host protein interactomes extracted from the STRING Disease database and imported into Cytoscape software for analysis. Significantly enriched GO terms shared between individual viral diseases and AD were plotted as a chord diagram and table (Fig. 9A) revealing substantial overlap. Functional enrichment of disease associated proteins yielded more than four thousand GO terms within the “Biological Process” and “Molecular Function” categories significantly associated with AD. Similar functional enrichment performed across 11 viral infections and the disease process of neuroinflammation demonstrated convergence with AD approaching 25% overlap in several viral pathogens with similar levels of convergence on neuroinflammation (Fig. 9). Additionally, pathway analysis performed on the compiled network of over 80 nodes (representing either genes/proteins or host response processes) linked to viral-induced AD-related pathology, identified key modules responsible for driving the disease process of inflammation of the central nervous system summarized in Supplementary Tables 2 and illustrated in Fig. 9B.

Querying the Ingenuity Pathway Analysis (IPA) Database (Qiagen) on the network composed of viral host response nodes (Supplementary Table 2) revealed neuroinflammation and pathogen-induced cytokine storm as the top common canonical pathways, with “inflammatory response” emerging as the most enriched disease function, encompassing over half of the total network nodes. These outcomes likely reflect shared upstream drivers across infections such as pattern recognition receptors including CGAS-STING and NOD1/2. Once activated, these cascades can trigger neurodegenerative pathology through downstream pathways involving S100 proteins, zinc homeostasis, and glucocorticoid signaling [76, 122, 123]. Importantly, this viral infection-response network is significantly enriched in nodes involved in neurological disease processes, including “inflammation of the central nervous system”. These processes encompass activation of phagocytes and leukocytes, autoimmune neurological disorder, and gliosis, each reported to be associated with Aβ and/or Tau pathology in 8 out of the 11 viral infections examined.

Collectively, protein-protein interaction (ppi) networks and pathway mapping approaches integrate protein datasets from experimental studies and literature to uncover key molecular hubs, such as MAPK, NF-κB, and PI3K-Akt, that viruses commonly hijack and also lie at the core of neurodegenerative signaling. Bioinformatic tools, predictive models and Public databases (VirHostNet, HPIDB, and VirusMentha) have greatly expanded the catalog of virus-host interactions. Machine-learning tools such as DeepViral and multitask transfer learning frameworks further predict novel PPIs using sequence homology and structural motifs, revealing that many viral proteins mimic host regulatory elements to enable neuronal entry and manipulation of autophagy and apoptosis.

Integrating genetic susceptibility and transcriptomic profiling offers another lens for investigating the molecular links between neurotropic viral infections and AD. Genetic predispositions, particularly the APOE4 allele amplify the effects of viral infections on Aβ deposition and tau pathology. Transcriptomic analyses across brain regions have identified molecular subtypes of AD, some enriched for antiviral response genes such as MA MDC2, which is highly expressed in microglia during neurotropic virus infection. Functional enrichment and network-convergence analyses of virus-host PPI networks consistently highlight biological processes including immune activation, oxidative stress, and synaptic dysfunction.

In sum, these approaches reveal that mechanisms central to viral pathogenesis are also key drivers of AD progression, supporting the concept of a shared molecular architecture. This convergence opens the opportunities for identifying early biomarkers and developing targeted therapeutic interventions that disrupt key interaction hubs shared between viral infections and AD pathology.

### Implications of viruses as exposomes for AD/ADRD: translational roadmap

Viruses as exposomes for AD/ADRD create meaningful opportunities for translational and interventional research. The mechanistic links summarized in this review highlight the potential to identify new biomarkers and therapies. Table 2 presents a cross-cutting feasibility and risk matrix outlining principal risks and clinical feasibility across candidate pathways. Our review organizes these targets in three tiers that represent short-term (high, medium) and long-term opportunities, as described below.

#### Tier1-high-priority, near-term feasible targets

Tier 1 targets represent the most immediately actionable pathways connecting viral exposomes to AD and ADRD. The strongest rationale centers on the NLRP3 inflammasome and the IL-1β-driven neuroinflammatory cascade, both activated by multiple neurotropic viruses including HSV-1 and SARS-CoV-2, and mechanistically linked to tau phosphorylation and impaired Aβ clearance [41, 48, 124]. In HSV-1–infected models, pharmacologic inhibition of NLRP3 reduces Aβ plaque burden and improves cognition, demonstrating translational viability [41]. In parallel, HSV-1 suppressive antiviral therapy emerges as a compelling candidate. Recurrent HSV-1 reactivation increases Aβ1–42 production, promotes tau splicing abnormalities via ICP27, activates NLRP3, disrupts BBB integrity, and worsens cognitive outcomes [29, 37, 41, 47, 125]. Given the widespread availability and safety of oral antivirals (e.g., valacyclovir), suppressive therapy trials in seropositive older adults or MCI patients are both feasible and mechanistically grounded [109, 111]. Finally, BBB stabilization warrants Tier 1 prioritization due to convergent viral effects on endothelial tight junctions, MMP-2/9 upregulation, immune-cell trafficking, and MFSD2A expression—particularly notable in SARS-CoV-2 infection [75, 87, 126–128]. BBB dysfunction acts as an amplifier of peripheral cytokine spillover and chronic neuroinflammation, positioning BBB-directed interventions as a critical early therapeutic entry point [124, 126].

**Table 2:** Cross-cutting feasibility and risk matrix

Target/Approach	Mechanistic Rationale (exposome → AD)	Key Biomarkers/Enrichment	Principal Risks	Translational Implication & Clin Feasibility (CF)
NLRP3/IL-1β/TNF-α axis inhibition	Convergent activation by HSV-1, SARS-CoV-2, CMV; NLRP3 drives tauopathy and impaired Aβ clearance	CSF/plasma IL-1β, IL-18, ASC/caspase-1; PET-microglia; fluid p-tau/Aβ	Infection susceptibility; readout specificity	*Platform anti-inflammatory* benefit across viruses; rapid pilot in MCI with neuroinflammation, CF: High (repurposing small molecules)
HSV-1 suppressive antivirals	APP/Aβ shifts, tau splice disruption, NLRP3 activation; reactivation worsens pathology	HSV-1 PCR/serology/reactivation markers; plasma/CSF Aβ42/40, p-tau181/217	Modest cognitive gain if late; adherence	Precision prevention in seropositive, APOE4^± older adults, CF: High-moderate
BBB stabilization	BBB leak amplifies cytokine ingress; MFSD2A loss in COVID-brains; MMPs up	DCE-MRI K’trans, CSF/serum albumin; MMP-9, ICAM-1/VCAM-1	Agent immaturity; systemic effects	Adjunct to antiviral/anti-inflammatory to break feed-forward loops, CF: moderate
Spike–APP/ACE2 axis modulation	Spike binds APP; ACE2 loss promotes Aβ/tau and inflammation; long-COVID cognitive risk	Post-COVID MCI; amyloid/tau PET; spike/APP engagement signatures	Variant biology; RAS side effects	Post-infectious secondary prevention trials, CF: moderate
CMV suppression / immune retraining	Tau-mimicry (UL131/IRL4), S396 tau; IFN-γ/TNF-α surges; MMP-9	CMV serology/pp65 antigenemia; tau T-cell cross-reactivity; p-tau/Aβ	Antiviral toxicity, uncertain effect size	Time-limited “reactivation-window” suppression; mechanistic PoC, CF: moderate
HIV CNS-optimized ART + anti-inflammatory	Tat/gp120 → ↑Aβ, ↓clearance, tau imbalance; HAND overlaps AD	CSF Tat/gp120; neuroinflammatory panels; amyloid/tau	Drug interactions; HAND heterogeneity	Aging PWH trials for cognitive preservation, CF: moderate
Seasonal vaccination (flu/COVID-19)	Prevents inflammatory hits that seed Aβ/tau cascades; epidemiologic risk reduction	Pre/post vaccine neuroinflammatory markers; cognitive composites	Confounding; indirect mechanism	Population-level exposome reduction strategy, CF: High (implementation)
HSV/CMV therapeutic vaccines	Reduce reactivation load over lifespan	Neutralizing Ab, T-cell breadth; exploratory neuro-biomarkers	Long timelines; unclear CNS correlates	Long-range prevention for high-risk genotypes, CF: Low-moderate (R&D)

#### Tier 2-medium-priority, mechanistically targeted opportunities

Tier 2 targets address viral mechanisms with strong biological plausibility but with either narrower population applicability or greater translational uncertainty. SARS-CoV-2–specific interventions, including modulation of ACE2 signaling and blockade of spike–APP interactions, fall within this tier: SARS-CoV-2 downregulates ACE2 neuroprotective activity, increases Aβ42 and plaque burden, perturbs tau-related pathways, and elevates inflammatory cytokines [79, 81–83, 129]. Although these mechanistic data are compelling, neuro-directed therapeutics remain in early development [78, 130]. CMV-directed strategies also merit Tier 2 classification; CMV reactivation induces chronic IFN-γ and TNF-α responses, drives MMP-9–mediated BBB disruption, and exhibits a distinctive molecular mimicry mechanism (UL131/IRL4) that cross-reacts with tau and promotes pathogenic phosphorylation at S396 [65, 68, 73, 74]. However, antiviral agents such as valganciclovir present toxicity concerns in older populations [67, 69]. Likewise, in HAND, HIV proteins Tat and gp120 accumulate in the CNS despite suppressive ART, where they promote Aβ42 aggregation, impair clearance, dysregulate Tau isoforms, and activate neurotoxic cytokine cascades [55, 61, 131, 132]. Optimizing ART regimens and pairing them with anti-inflammatory adjunct therapies may reduce HAND-to-AD progression risk, but require careful management of safety and drug–drug interactions [54, 124].

#### Tier 3- strategic, long-term preventive approaches

Tier 3 approaches focus on population-level prevention and future-oriented vaccines aimed at reducing cumulative viral exposures that contribute to AD-related neuroinflammation. Seasonal influenza vaccination is supported by both epidemiologic and experimental evidence linking vaccination (or reduced infection burden) to lower dementia risk and attenuated inflammatory “hits” capable of NMDAR-mediated Aβ release, LRRK2-dependent tau phosphorylation, and microglial activation [31, 33, 92, 133–135]. Similarly, COVID-19 vaccination may reduce long-COVID–associated neuroinflammatory phenotypes that resemble AD-like molecular signatures [83, 136]. These levers are safe, scalable, and already widely implemented. Tier 3 also includes HSV-1 and CMV vaccine development, which carries high long-term value due to the lifelong latency and reactivation cycle of these viruses. By suppressing reactivation, such vaccines could reduce recurrent neuroinflammation, APP dysregulation, Tau pathology, and BBB permeability [65, 69, 108, 109]. Although promising, these vaccines remain under development, and the absence of validated CNS-specific clinical endpoints places them into a long-range strategic category [112, 137].

Overall, across all domains, key risks include inadequate modeling of chronic infection biology, exposure misclassification in clinical datasets, population heterogeneity, and confounding by genetic modifiers such as APOE4 [96]. These challenges can be addressed by prioritizing human-based experimental systems, standardizing viral detection and biomarker assays, and applying rigorous causal inference frameworks [113, 138]. Ultimately, rapid progress hinges on achieving mechanistic clarity: human-relevant experimental evidence linking viral pathways to AD; biomarker-driven clinical studies capturing viral reactivation and downstream inflammation; and computational frameworks that unify these layers into actionable targets [107, 114]. Collectively, these efforts can transform the infectious hypothesis from a conceptual model into a foundation for targeted diagnostics, preventive strategies, and mechanism-guided therapeutics [96].

### Overcoming the gaps & actionable future directions

AD remains a major unmet medical need. Converging evidence indicates that latent, reactivating, and persistent viral infections can act upstream of neuroinflammation, Aβ dysregulation, Tau pathology, and accelerated cognitive decline. Progress requires coordinated experimental, clinical, and computational strategies to clarify how neurotropic viral exposures shape AD pathogenesis. ^26,28,34,115^

#### Experimental gaps and required platforms

Current mechanistic understanding is severely constrained by major shortcomings in existing models: conventional mice do not recapitulate human-specific viral latency or physiologic reactivation cycles, and do not reflect the decades-long, low-grade chronic exposure that characterizes the human viral exposome [120, 121, 139]. Acute, high-dose infection models distort natural disease dynamics, while post-mortem human studies captures only static snapshots of end-stage pathology, lacking temporal or causal resolution [24]. These limitations prevent identification of how lifelong viral exposures interact with aging, genetics, and immune dysregulation to shape neurodegenerative trajectories [28, 118]. A few next-generation experimental platforms can address these barriers.

First, human brain organoids, especially those containing microglia and functional BBB interfaces can be exploited, which allow viruses to establish authentic persistent states and enable physiologic reactivation using stressors relevant to human biology [28, 118]. Second, next-generation humanized mouse models support human viral tropism, immune responses, and latency–reactivation cycles that more faithfully mirror human infection dynamics [119, 139]. Third, organoid-on-chip and multi-organ microfluidic systems extend these capabilities by modeling multi-tissue viral interactions, circulating immune components, and long-term, low-dose exposures [118, 139]. for dissecting tissue-tissue crosstalk under physiologic stressors [15, 29, 36, 100, 138].

Together, these platforms recreate the dimensions of viral biology essential for studying progressive neurodegeneration but inaccessible in conventional models. These platforms would interrogate convergent AD nodes, including NLRP3 inflammasome activation, autophagy and lysosomal failure, APP–viral protein interactions, BBB breakdown, and genetic modifiers (e.g., APOE4) that amplify amyloidogenic/inflammatory responses [28, 29, 36, 80, 116, 140]. Human-relevant platforms are expected to accelerate identification of mechanism-validated targets, early biomarkers, and preventive or therapeutic strategies that reflect authentic infection biology [15, 121–123].

#### Clinical directions

There are several actionable future directions in clinical arena. These include: i) *Shift to prospective*,* lab-confirmed cohorts.* Move beyond retrospective coding to longitudinal, laboratory-confirmed viral exposome cohorts with standardized biomarkers/imaging [2, 71, 141], including: (a) Viral assays: HSV-1/CMV PCR/antigenemia, serologic avidity; cytokines; (b) Neuro-biomarkers: Aβ42/40, p-tau species; microglial imaging; and (c) Genetics: APOE4 and innate immune modifiers for stratification [108, 122] ii) *Pragmatic clinical panels*. Implement reactivation plus inflammation panels (HSV-1 markers, CMV pp65, IL-1β/IL-18, p-tau217/231, microglial PET, BBB metrics: DCE-MRI; CSF/serum albumin) to detect virus-driven pathology early and enrich trials [15, 48, 54, 125] ; iii) *Mechanism-anchored early-phase trials (MCI).* Enroll MCI/high-risk adults enriched for reactivation, neuroinflammatory signatures, or APOE4; prioritize NLRP3/IL-1β modulation, HSV-1 suppression, CMV-window antivirals, BBB restoration, and spike–APP/ACE2 modulation; use biomarker endpoints (p-tau217, IL-1β/IL-18, DCE-MRI, microglial PET) [47, 54, 84, 85, 120, 124, 136] ; iv) *Population-level prevention.* Leverage influenza/COVID-19 vaccination to reduce systemic inflammatory “hits”; embed pre/post biomarker sub-studies (cytokines, p-tau217, DCE-MRI) to test modifiability of virus-driven neuroinflammation [38–40, 106]; v) *Viral exposome scoring.* Create composite scores integrating serostatus, reactivation frequency, cytokines, co-infection clusters to identify high-risk subgroups and support mechanism-based stratification (NLRP3/Aβ/tau axes) [21, 121, 124]. In sum, by providing actionable, translatable insights into human-specific infection biology, these directions position the field to identify modifiable risk pathways, accelerate biomarker discovery for early detection, and lay the foundation for antiviral, immunomodulatory, and preventive interventions that could meaningfully alter disease trajectory [119].

#### Computational and machine learning approaches

With the advances computational science, particularly machine learning, several actionable directions are recommended. (i) Unify knowledge graph (KG). Build an integrated virus–host–AD KG combining PPIs, transcriptomics, exposures, genetics (APOE4/TREM2), imaging (microglial PET, DCE-MRI), and fluid biomarkers (p-tau217, Aβ42/40, IL-1β/IL-18), centered on convergent hubs (MAPK, NF-κB, PI3K-Akt, cGAS–STING, NLRP3). (ii) Harmonize data standards and FAIR (Findable, Accessible, interoperable, Reusable) pipelines for multi-site integration. Standardize assays, units, ontologies (primary infection, latency, and reactivation) and metadata to enable cross-cohort meta-analysis, correct misclassification, and link molecular readouts to endpoints [15, 21, 121, 124]; (iii) *Fuse signals across scales using multimodal representation learning.* Use GNNs and late-fusion to produce patient embeddings combining NLRP3 activity, BBB leak, HSV-1/CMV reactivation; encode temporal dynamics (cumulative hits, lagged effects) for lifetime exposure [15, 21, 54, 121, 124]; (iv) *Separate signal from confounding via causal inference pipelines*. Apply propensity scores, marginal structural models, instrumental variables, negative controls, and explicit temporality tests to show reactivation precedes inflammation/tau changes; embed DAGs within the KG to distinguish drivers from correlates [21, 41, 54, 78, 142]; (v) *Exploit trial-readiness analytics: enrichment*,* endpoints*,* and digital twins.* Enable enrichment (reactivation, NLRP3, BBB leak) and biomarker endpoints (p-tau217, IL-1β/IL-18, DCE-MRI K’trans, microglial PET); use digital twins and KG-anchored synthetic controls for adaptive designs and smaller, mechanism-focused trials [15, 54, 78, 121, 124]; (vi) *U*se *privacy-preserving*,* federated learning across institutions.* Train models across diverse sites without sharing raw PHI; federated KG updates and model averaging support generalizable exposome–AD signatures while preserving privacy. (vii) *Improve robustness*,* interpretability*,* and mechanism-to-model traceability.* Stress-test for missingness and batch effects; provide pathway-level attribution (e.g., NLRP3/NF-κB), counterfactuals, and KG-anchored saliency to ensure mechanism-to-model traceability [54, 121, 142]; and (viii) *Establish benchmarking and reproducibility framework.* Create shared datasets (harmonized viral assays, biomarker panels, imaging) and standard metrics (reactivation prediction, inflammasome treatment-effect, BBB leak calibration); follow open, version-locked protocols mirroring your systems-bioinformatics workflows [15, 21, 78, 121].

In closing, translating the emerging viral-exposome paradigm into actionable AD interventions requires a coordinated developmental roadmap spanning experimental, clinical, and computational domains. Experimentally, progress depends on human-relevant models including iPSC-derived brain organoids with microglia and BBB interfaces, organoid-on-chip systems, and next-generation humanized mice to faithfully recapitulate viral latency, reactivation, and chronic low-grade exposure, enabling mechanistic dissection of convergent pathways such as NLRP3 inflammasome activation, autophagy failure, APP–viral protein interactions, and BBB compromise. Clinically, advancement requires establishing prospective, laboratory-confirmed viral exposome cohorts with standardized viral assays, neuroinflammatory panels, Aβ/tau biomarkers, and microglial/BBB imaging, integrated with genetic risk factors (e.g., APOE4) to strengthen causal inference and refine risk stratification. These cohorts should support mechanism-anchored early-phase trials in high-risk or mild cognitive impaired populations, prioritizing targets such as NLRP3, IL-1β, HSV-1/CMV suppression, BBB-repair therapeutics, and post-viral (e.g., SARS-CoV-2) host-interaction disruptors, using biomarker endpoints rather than late symptomatic outcomes. Computationally, progress hinges on integrated virus–host–AD knowledge graphs, harmonized FAIR data pipelines, multimodal representation learning, and causal inference frameworks capable of distinguishing true viral drivers from confounding processes. Together, these pathways provide the foundation for precision diagnostics, exposome-informed prevention, and targeted mechanism-guided therapeutics, accelerating the path from association to intervention in AD and ADRD.

## Electronic Supplementary Material

Below is the link to the electronic supplementary material.

Supplementary Material 1.

## Data Availability

No new datasets were generated during the current study.

## Abbreviations

AD: Alzheimer’s disease
ADRD: Alzheimer’s disease–related dementias
HSV-1 / HSV1: Herpes simplex virus type 1
HIV: Human immunodeficiency virus
CMV: Cytomegalovirus
SARS-CoV-2: Severe acute respiratory syndrome coronavirus 2
Aβ: Amyloid-β
Tau: Microtubule-associated protein tau
BBB: Blood–brain barrier
APP: Amyloid precursor protein
BACE1: β-site APP-cleaving enzyme 1
NFT: Neurofibrillary tangles
PD: Parkinson’s disease
NDD: Neurodegenerative disease
ALS: Amyotrophic lateral sclerosis
VAS: Vascular dementia
MS: Multiple sclerosis
NLRP3: NOD-, LRR-, and pyrin domain-containing protein 3
IL-1β: Interleukin-1 beta
TNF-α: Tumor necrosis factor alpha
IFN-γ: Interferon gamma
iNOS: Inducible nitric oxide synthase
NO: Nitric oxide
KEGG: Kyoto Encyclopedia of Genes and Genomes
GO: Gene ontology
PPI: Protein–protein interaction
IPA: Ingenuity Pathway Analysis
CSF: Cerebrospinal fluid
APOE4: Apolipoprotein E epsilon-4 allele
MAPT: Microtubule-associated protein tau gene
GSK-3β: Glycogen synthase kinase-3 beta
PP2A: Protein phosphatase 2 A
ICP: Infected cell protein
BECN1: Beclin-1
ICAM-1: Intercellular adhesion molecule-1
HAND: HIV-associated neurocognitive disorder
ART: Antiretroviral therapy
ROS: Reactive oxygen species
CDK5: Cyclin-dependent kinase 5
ACE2: Angiotensin-converting enzyme 2
BDNF: Brain-derived neurotrophic factor
MFSD2A: Major facilitator superfamily domain containing 2 A
UPR: Unfolded protein response
ER: Endoplasmic reticulum
LRRK2: Leucine-rich repeat kinase 2
NMDA / NMDAR: N-methyl-D-aspartate receptor
EBV: Epstein–Barr virus
ZIKV: Zika virus
VZV: Varicella–zoster virus
HHV-6 / HHV-7: Human herpesvirus-6 / -7CI: Mild cognitive impairment
DCEMRI: Dynamic contrast-enhanced magnetic resonance imaging
PET: Positron emission tomographyKG: Knowledge graph
