# Supplementary material for: Convergence of neuroinflammation across major neurotropic viral exposomes in AD and ADRD
Source: J Neuroinflammation. 2026 May 29;23:265. doi: 10.1186/s12974-026-03876-2 (PMC13430881; doi:10.1186/s12974-026-03876-2)
Supplement: Supplementary file 1 — Supplementary Material 1. [file 12974_2026_3876_MOESM1_ESM.docx]

**Supplementary data**

Title: Neuroinflammation Convergence Among the Major Neurotropic Viral Exposomes in AD and ADRD: A Systematic Review

Jamile Harmouch et al, J of Neuro Inflammatation.

Corresponding authors: Shyam S Mohapatra([smohapat@usf.edu](mailto:smohapat@usf.edu)) and

Subhra Mohapatra (smohapa2@usf.edu)

**Supplementary Table 1. The relevant databases used in the study.**

| Accession ID’s used for creating networks of virus related proteins | NIV:DOID 50192, HSV:DOID 8566, hsa05168, HP:0012302, MAP05168 ZIKV: DOID0060478, RABV: DOID:11260, JEV: DOID 10844, HIV: HAS-162906, DOID526, EFO:0000180,map-162906,map-167161, map-167169, map-162587,EFO0000764 SARS-CoV-2: HSA9694516, DOID 0080600, map-9694516, map-9705683, WP5098, map-9705671, WP5039, WNV:DOID 2365, DOID:2366 CMV: EFO_007037, map01563. Influenza: DOID:8469, HSA-168255, hsa05164, map-168255, RSV: DOID:1273. neuroinflammation: GO:0150076, GO:0150078, WP4919, HP:0033429. |
| --- | --- |
| Evidence of involvement of genes/proteins in the viral host response presented in relevant publications by using the “Geneset by Pathway / Process / Disease / Publication” search feature of STRING | Proteins from the following publications describing viral host response were imported: NIV: PMID: 33328346, PMID: 27622505, PMID: 31006350, PMID: 26105519, PMID: 34785771, JEV: PMID: 37086856, PMID: 36560690, PMID: 33724154, PMID: 25188232, PMID: 21371334. RABV: PMCID: PMC5124403, PMID: 21994572, PMID: 26198243. ZIKV: PMID: 31546825, PMID: 31531178, PMID: 33810028, PMID: 36016430, PMID: 37684223, PMID: 27610098, PMID: 37891676, PMID: 29430005, PMID: 35371036, PMID: 28325921. HSV: PMID:32708188, PMID:31114761, PMID:34895058, PMID:34830340, PMID:35793266, PMID:36429022, PMID:36094100, PMID:36839582, PMID:34281386. CMV: PMID: 34200083, PMID: 33096622, PMID: 31921100, PMID: 33567734, PMID: 32296651, HIV: PMID:37408185, PMID:36927791, PMID:36639783, SARS-CoV-2: PMID:33376956, PMID:32531256, PMID:37404823, WNV: PMID: 24178712, PMID: 23544010, PMID: 36992514, PMID: 38047068, PMID: 27211830. Influenza: PMID:32674269, PMID:34372568, RSV: PMID:34960746, PMID:35216012 |

**Supplementary Table 2.** Convergent Neuroinflammatory and Neurodegenerative Pathways Across Diverse Viral Infections: A Comparative Analysis of Amyloid Pathology, Tau Dysfunction, Blood-Brain Barrier Disruption, and Inflammatory Markers.

| **Category** | | **HSV** | **HIV** | **CMV** | **SARS-CoV-2** | **RABV** | **ZIKV** | **JEV** | **WNV** | **NiV** | **Influenza** | **RSV** |
| --- | --- | --- | --- | --- | --- | --- | --- | --- | --- | --- | --- | --- |
| **Latency** | | Yes | Yes | Yes | No | No | No | No | No | Yes | No | No |
| **Family** | | *Herpesviridae* | *Retroviridae* | *Herpesviridae* | *Coronaviridae* | *Rhabdoviridae* | *Flaviviridae* | *Flaviviridae* | *Flaviviridae* | *Paramyxoviridae* | *Orthomyxoviridae* | *Pneumoviridae* |
| **Amyloid pathology** | | APP processing, ICP 8 interaction, caspase-3 activation reduced microglial phagocytosis[1] | Tat and gpl20 interact with  APP processing, ↑ β-sheet formation, inhibit neprilysin, and reduce Aβ clearance[2] | Aβ acts as an AMP.  Chronic infection causes Aβ  accumulation[3] | Via ACE2 inhibition, spike-APP interaction, ↑ in IFITM3, ARRB1, RAC1, and  LGALS3[4] | Not Reported | ↑ Aβ via BACE1[5] | ↑ Aβ, ↑ APP[6] | ↑ APP, ↑Aβ 40, ↑ Aβ 42[7] | Not Reported | Inflammation-mediated Aβ plaque formation, HA-mediated Aβ seeding, NMDAR-mediated release[8] | Not Reported |
| **Tau pathology** | | Alters MAPT slicing via ICP27[9] | ↑ in Tau 3R via tat-induced SC35  phosphorylation. gp120  induces TNF[10,11] | Cross reactive T cells against Tau (UL131  and IRL4). S396 phosphorylation-induced NFT formation[12] | Via BDNF and SRSF1  reduction. ↑ in Ca2+ dependent enzymes and FKBP5[13,14] | Not Reported | ↑ p-Tau via GSK-3α/β[5] | Regulation of miR-125b-5p[15] | Abnormal tau phosphorylation, NFT[7] | Not Reported | LRRK2-dependent Tau phosphorylation, NMDAR-related Tau-dependent toxicity[16] | Not Reported |
| **Markers of BBB disruption** | ↑ ICAM-1, MMP-2, MMP-9, VCAM-1[17] | | Tat-mediated damage, ↑ MMP-2, MMP-9, ICAM-1, VCAM-1, Tight junctions degradation[18] | ↑ MMP-9, Tight junctions disruption [19] | MFSD2A reduction, tight junctions'disruption[20] | ↓ Tight junction proteins[21] | Further investigations needed[21-23] | ↑ JAM, ↑ ICAM-1, ↑ CINC-1, ↓ Tight junctions, ↓ Claudin-1, ↓ Claudin-5, ↓ ZO-1/occludin[21,23] | ↑ MMPs, ↓ Tight junctions, ↓ TEER[21,24] | Endothelial destruction, Microinfarction, Vasculitis, Thrombosis[21,23] | No disruption detected | Immune cells infiltration, ↑ Evans Blue extravasation, impaired astrocyte function[25-27] |
| **Markers of Neuro-inflammation** | NLRP3, IL-1β, and chronic microglial activation. Associated with ICP34.5[28,29] | | Gp120 and Tat- induced proinflammatory cytokines release and oxidative stress; ↑IFN-γ, ΙL-6, IL-8, IL-1, COX-2, TNF-α,IL-1β, ROS[30,31] | pp65-induced pro-  inflammatory cytokines release; ↑IFN-γ, IL-2, IP-10, TNF-α [32] | Microglia activation,  NLRP3  inflammasome, pro-inflammatory cytokines, ROS.  ↑ in IFI16, ESR1, TMEM119, caspase-1, NMDAR2[21,23] | TLR4, TLR3, IFN-1, CCL5, IP-10, MCP-1, IL-6, RIG-I, IL-1, CXCL10, AIM2, MDA5, BAX, BCL2, Caspases, ROS[33] | TLR3, IL-1β, NF-κB, TNF-α, IL-6, IL-1α, IL-4, IL-10, IL-8, MCP-1, IFN-β, MIP-1β, NLRP3 inflammasome, ROS, MDA, iNOS, NO, ER stress[21-23] | CLEC5A, IL-1β, TNF-α, IL-1α, IL-8, IL-10, caspase-3, AP-1, NF-κB, MCP-1, IL-1, IL-6, IL-8, TNF-α, MMP, O₂⁻, NO, PERK, ER stress[21,23] | TLR3, TLR7, IFN-1, IRF3, BAX, cleavage of PARP, caspase-3, caspase-8, caspase-9, pro-inflammatory cytokines, chemokines[21,23,24] | IL-6, IL-8, IL-1, MCP-1, TNF-α, IP-10, IL-1β, IFN-β, IL-1αIL-6, IL-8, IL-1, MCP-1, TNF-α, IP-10, IL-1β, IFN-β, IL-1α[23] | Microglia activation,IL-1β, TNF-α, IL-6 , influenza-related desialylation, CD200 and CX3CL1 downregulation[34,35] | Microglia activation, IL-6, IL-8, CCL2, CCL4, NO, IL-1β TNF-α, f IL-4, IL-10,GFAP, downregulation of CD200 and CX3CL1[25,36,37] |

**Supplementary References:**

1. Gao M, Knipe DM. Potential role for herpes simplex virus ICP8 DNA replication protein in stimulation of late gene expression. *J Virol*. May 1991;65(5):2666-75. doi:10.1128/JVI.65.5.2666-2675.1991

2. Hategan A, Masliah E, Nath A. HIV and Alzheimer's disease: complex interactions of HIV-Tat with amyloid beta peptide and Tau protein. *J Neurovirol*. Oct 2019;25(5):648-660. doi:10.1007/s13365-019-00736-z

3. Allnutt MA, Johnson K, Bennett DA, et al. Human Herpesvirus 6 Detection in Alzheimer's Disease Cases and Controls across Multiple Cohorts. *Neuron*. Mar 18 2020;105(6):1027-1035 e2. doi:10.1016/j.neuron.2019.12.031

4. Bhardwaj T, Gadhave K, Kapuganti SK, et al. Amyloidogenic proteins in the SARS-CoV and SARS-CoV-2 proteomes. *Nat Commun*. Feb 20 2023;14(1):945. doi:10.1038/s41467-023-36234-4

5. Lee SE, Choi H, Shin N, et al. Zika virus infection accelerates Alzheimer's disease phenotypes in brain organoids. *Cell Death Discov*. Apr 2 2022;8(1):153. doi:10.1038/s41420-022-00958-x

6. Yin R, Yang L, Hao Y, et al. Proteomic landscape subtype and clinical prognosis of patients with the cognitive impairment by Japanese encephalitis infection. *J Neuroinflammation*. Apr 4 2022;19(1):77. doi:10.1186/s12974-022-02439-5

7. Kobayashi S, Yoshii K, Phongphaew W, et al. West Nile virus capsid protein inhibits autophagy by AMP-activated protein kinase degradation in neurological disease development. *PLoS Pathog*. Jan 2020;16(1):e1008238. doi:10.1371/journal.ppat.1008238

8. Hosseini S, Michaelsen-Preusse K, Schughart K, Korte M. Long-Term Consequence of Non-neurotropic H3N2 Influenza A Virus Infection for the Progression of Alzheimer's Disease Symptoms. *Front Cell Neurosci*. 2021;15:643650. doi:10.3389/fncel.2021.643650

9. Ijezie EC, Miller MJ, Hardy C, et al. Herpes simplex virus-1 infection alters microtubule-associated protein Tau splicing and promotes Tau pathology in neural models of Alzheimer's disease. *Brain Pathol*. Sep 2025;35(5):e70006. doi:10.1111/bpa.70006

10. Vijayan M, Yin L, Reddy PH, Benamar K. Behavioral Evidence for a Tau and HIV-gp120 Interaction. *Int J Mol Sci*. May 15 2022;23(10)doi:10.3390/ijms23105514

11. Gonzalez J, Wilson A, Byrd D, Cortes EP, Crary JF, Morgello S. Neuronal accumulation of hyperphosphorylated tau protein predicts stable memory impairment in people living with HIV. *AIDS*. Jul 1 2023;37(8):1247-1256. doi:10.1097/QAD.0000000000003556

12. Lurain NS, Hanson BA, Martinson J, et al. Virological and immunological characteristics of human cytomegalovirus infection associated with Alzheimer disease. *J Infect Dis*. Aug 15 2013;208(4):564-72. doi:10.1093/infdis/jit210

13. Green R, Mayilsamy K, McGill AR, et al. SARS-CoV-2 infection increases the gene expression profile for Alzheimer's disease risk. *Mol Ther Methods Clin Dev*. Dec 8 2022;27:217-229. doi:10.1016/j.omtm.2022.09.007

14. Orsini F, Bosica M, Martucci A, et al. SARS-CoV-2 Nucleocapsid Protein Induces Tau Pathological Changes That Can Be Counteracted by SUMO2. *Int J Mol Sci*. Jun 28 2024;25(13)doi:10.3390/ijms25137169

15. Lewis A, Frontera J, Placantonakis DG, et al. Cerebrospinal fluid in COVID-19: A systematic review of the literature. *J Neurol Sci*. Feb 15 2021;421:117316. doi:10.1016/j.jns.2021.117316

16. Cousins O, Schubert JJ, Chandra A, et al. Microglial activation, tau and amyloid deposition in TREM2 p.R47H carriers and mild cognitive impairment patients: a multi-modal/multi-tracer PET/MRI imaging study with influenza vaccine immune challenge. *J Neuroinflammation*. Nov 21 2023;20(1):272. doi:10.1186/s12974-023-02945-0

17. Liu H, Qiu K, He Q, Lei Q, Lu W. Mechanisms of Blood-Brain Barrier Disruption in Herpes Simplex Encephalitis. *J Neuroimmune Pharmacol*. Jun 2019;14(2):157-172. doi:10.1007/s11481-018-9821-6

18. Sun Y, Cai M, Liang Y, Zhang Y. Disruption of blood-brain barrier: effects of HIV Tat on brain microvascular endothelial cells and tight junction proteins. *J Neurovirol*. Dec 2023;29(6):658-668. doi:10.1007/s13365-023-01179-3

19. Kawasaki H, Kosugi I, Meguro S, Iwashita T. Pathogenesis of developmental anomalies of the central nervous system induced by congenital cytomegalovirus infection. *Pathol Int*. Feb 2017;67(2):72-82. doi:10.1111/pin.12502

20. Jakhmola S, Indari O, Chatterjee S, Jha HC. SARS-CoV-2, an Underestimated Pathogen of the Nervous System. *SN Compr Clin Med*. 2020;2(11):2137-2146. doi:10.1007/s42399-020-00522-7

21. Wongchitrat P, Chanmee T, Govitrapong P. Molecular Mechanisms Associated with Neurodegeneration of Neurotropic Viral Infection. *Mol Neurobiol*. May 2024;61(5):2881-2903. doi:10.1007/s12035-023-03761-6

22. Leda AR, Bertrand L, Andras IE, El-Hage N, Nair M, Toborek M. Selective Disruption of the Blood-Brain Barrier by Zika Virus. *Front Microbiol*. 2019;10:2158. doi:10.3389/fmicb.2019.02158

23. Al-Obaidi MMJ, Bahadoran A, Wang SM, Manikam R, Raju CS, Sekaran SD. Disruption of the blood brain barrier is vital property of neurotropic viral infection of the central nervous system. *Acta Virol*. 2018;62(1):16-27. doi:10.4149/av_2018_102

24. Beltrami S, Rizzo S, Schiuma G, et al. West Nile virus non-structural protein 1 promotes amyloid Beta deposition and neurodegeneration. *Int J Biol Macromol*. May 2025;305(Pt 1):141032. doi:10.1016/j.ijbiomac.2025.141032

25. Bohmwald K, Soto JA, Andrade-Parra C, et al. Lung pathology due to hRSV infection impairs blood-brain barrier permeability enabling astrocyte infection and a long-lasting inflammation in the CNS. *Brain Behav Immun*. Jan 2021;91:159-171. doi:10.1016/j.bbi.2020.09.021

26. Raber J, Rhea EM, Banks WA. The Effects of Viruses on Insulin Sensitivity and Blood-Brain Barrier Function. *Int J Mol Sci*. Jan 25 2023;24(3)doi:10.3390/ijms24032377

27. Mora VP, Kalergis AM, Bohmwald K. Neurological Impact of Respiratory Viruses: Insights into Glial Cell Responses in the Central Nervous System. *Microorganisms*. Aug 20 2024;12(8)doi:10.3390/microorganisms12081713

28. Wang Z, Liu J, Han J, et al. Herpes simplex virus 1 accelerates the progression of Alzheimer's disease by modulating microglial phagocytosis and activating NLRP3 pathway. *J Neuroinflammation*. Jul 18 2024;21(1):176. doi:10.1186/s12974-024-03166-9

29. Harris SA, Harris EA. Molecular Mechanisms for Herpes Simplex Virus Type 1 Pathogenesis in Alzheimer's Disease. *Front Aging Neurosci*. 2018;10:48. doi:10.3389/fnagi.2018.00048

30. Lee YJ, Yeo IJ, Choi DY, et al. Amyloidogenic, neuroinflammatory and memory dysfunction effects of HIV-1 gp120. *Arch Pharm Res*. Jul 2021;44(7):689-701. doi:10.1007/s12272-021-01340-8

31. Mustafa M, Musselman D, Jayaweera D, da Fonseca Ferreira A, Marzouka G, Dong C. HIV-Associated Neurocognitive Disorder (HAND) and Alzheimer's Disease Pathogenesis: Future Directions for Diagnosis and Treatment. *Int J Mol Sci*. Oct 17 2024;25(20)doi:10.3390/ijms252011170

32. Westman G, Berglund D, Widen J, et al. Increased inflammatory response in cytomegalovirus seropositive patients with Alzheimer's disease. *PLoS One*. 2014;9(5):e96779. doi:10.1371/journal.pone.0096779

33. Agnello L, Ciaccio M. Neurodegenerative Diseases: From Molecular Basis to Therapy. *Int J Mol Sci*. Oct 25 2022;23(21)doi:10.3390/ijms232112854

34. Jurgens HA, Amancherla K, Johnson RW. Influenza infection induces neuroinflammation, alters hippocampal neuron morphology, and impairs cognition in adult mice. *J Neurosci*. Mar 21 2012;32(12):3958-68. doi:10.1523/JNEUROSCI.6389-11.2012

35. Falsey AR. Neurologic Complications of Influenza and Potential Protective Vaccine Effects. *Influenza Other Respir Viruses*. Mar 2025;19(3):e70071. doi:10.1111/irv.70071

36. Bohmwald K, Galvez NMS, Rios M, Kalergis AM. Neurologic Alterations Due to Respiratory Virus Infections. *Front Cell Neurosci*. 2018;12:386. doi:10.3389/fncel.2018.00386

37. Bohmwald K, Espinoza JA, Gonzalez PA, Bueno SM, Riedel CA, Kalergis AM. Central nervous system alterations caused by infection with the human respiratory syncytial virus. *Rev Med Virol*. Nov 2014;24(6):407-19. doi:10.1002/rmv.1813
